# Gut Seasons: Photoperiod Effects on Fecal Microbiota in Healthy and Cafeteria-Induced Obese Fisher 344 Rats

**DOI:** 10.3390/nu14030722

**Published:** 2022-02-08

**Authors:** Verónica Arreaza-Gil, Iván Escobar-Martínez, Manuel Suárez, Francisca Isabel Bravo, Begoña Muguerza, Anna Arola-Arnal, Cristina Torres-Fuentes

**Affiliations:** Nutrigenomics Research Group, Departament de Bioquímica i Biotecnologia, Universitat Rovira i Virgili, 43007 Tarragona, Spain; veronica.arreaza@urv.cat (V.A.-G.); ivan.escobar@urv.cat (I.E.-M.); manuel.suarez@urv.cat (M.S.); franciscaisabel.bravo@urv.cat (F.I.B.); begona.muguerza@urv.cat (B.M.); cristina.torres@urv.cat (C.T.-F.)

**Keywords:** gut microbiota, photoperiods, seasonal rhythms, obesity, cafeteria diet

## Abstract

Gut microbiota and biological rhythms are emerging as key factors in the modulation of several physiological and metabolic processes. However, little is known about their interaction and how this may affect host physiology and metabolism. Several studies have shown oscillations of gut microbiota that follows a circadian rhythmicity, but, in contrast, variations due to seasonal rhythms have not been sufficiently investigated yet. Thus, the goal of this study was to investigate the impact of different photoperiods, which mimic seasonal changes, on fecal microbiota composition and how this interaction affects diet-induced obesity development. To this aim, Fisher 344 male rats were housed under three photoperiods (L6, L12 and L18) and fed with standard chow diet or cafeteria diet (CAF) for 9 weeks. The 16S ribosomal sequencing of collected fecal samples was performed. The photoperiod exposure significantly altered the fecal microbiota composition under L18, especially in CAF-fed rats. Moreover, these alterations were associated with changes in body weight gain and different fat parameters. These findings suggest a clear impact of seasonal rhythms on gut microbiota, which ultimately translates into different susceptibilities to diet-induced obesity development. This is the first time to our knowledge that the photoperiod impact on gut microbiota composition has been described in an obesity context although further studies are needed in order to elucidate the mechanisms involved.

## 1. Introduction

Gut microbiota composition has been described as critical for the maintenance of homeostasis and metabolic function in the host [1]. Alterations in gut microbiota, also known as dysbiosis, may interfere with this balance, contributing to the development of metabolic diseases such as obesity and metabolic syndrome (MetS) [2]. Changes in dietary patterns are one of the most critical, modifiable factors that significantly alter the gut microbiota composition [3]. Thus, traditional diets such as the Mediterranean diet, which consists of high consumption of fiber and low consumption of sugar and fat, have long been associated with an increase in gut microbiota diversity and with a higher health status [4]. By contrast, Western-style diets have been shown to have strong effects on gut microbiota diversity and composition, often correlated with deleterious metabolic health effects [5]. For instance, the chronic consumption of a cafeteria diet significantly decreased gut bacterial diversity, reducing Firmicutes and increasing Bacteroidetes and Proteobacteria abundances, which were correlated with altered levels of plasma leptin and glycerol, as well as adipose tissue and liver inflammation, leading to the development of obesity and MetS [6].

In addition to diet, biological rhythms are emerging as a key factor to take into consideration when investigating gut microbiota changes. Indeed, several studies have shown oscillations of gut microbiota during 24 h cycles [7,8]. In the latest years, a growing body of evidence has shown that circadian rhythms can interact with nutrients, influencing several metabolic and physiological functions [9,10]. This relatively new field is described as “chrononutrition”. Hence, the food-intake pattern during the day has a significant influence on postprandial glucose, consequently affecting metabolism. The presence of these rhythms allows the organism to adjust to environmental factors such as changes in food availability or climatic variability, ensuring reproductive success and survival [11]. The effects of circadian rhythms on metabolism and physiology have been the most studied but those related to circannual rhythms are recently receiving increasing interest due to their important role in the regulation of physiological responses [12,13]. The synchronization between seasonal rhythms and physiological processes is driven by changes in the length of the daylight phase duration (photoperiods) [14,15]. The response to changes in photoperiods is encoded by the suprachiasmatic nucleus in the hypothalamus through the control of pineal melatonin production [15,16]. The melatonin signal communicates photoperiod information to a variety of targets throughout the body and brain, being therefore the hypothalamic–pituitary axis indispensable for the interaction between seasonal changes and both metabolic and physiological processes [17]. Thus, seasonal rhythms have been related to psychiatric disorders [18] and reproductive alterations in humans [19]. Furthermore, recent studies have shown that exposure of normal-weight rats to different photoperiods led to different metabolic changes suggesting that glucose- and lipid-related pathologies, such as obesity and MetS, could be influenced by light variations such as those observed in the different seasons [20]. However, little is known about the specific mechanisms involved. In fact, the effects of seasonal rhythms on gut microbiota which, as mentioned above, is one of the main links between diet and host metabolism, have not been sufficiently investigated yet.

In this regard, it has been shown that gut microbiota composition changes in both winter and summer due to seasonal variations in both the length of the daylight phase [21] and in dietary patterns [22]. Previous studies indicated that the relative abundance of certain bacteria differed for Siberian hamsters housed in long- versus short-day lengths [21,23]. In addition, seasonal variations in gut bacteria related to dietary changes were found in plateau pikas [24]. In another study in giant pandas, seasonal oscillations of gut microbiota and higher short chain fatty acid (SCFA) production in the shoot-eating season were observed [25]. In addition, seasonal changes including an increase in the breeding season of the relative abundance of gut bacteria related to lipid metabolism, carbohydrate metabolism, and nucleotide metabolism were observed in wild ground squirrels [26]. Significant seasonal oscillations in structure and function of gut bacteria were also found in forest and alpine musk deer [27]. In particular, a decrease in both the relative abundance of Firmicutes and the ratio of Firmicutes to Bacteroidetes, as well as an increase in Bacteroidetes, was observed in summer compared to winter. Authors concluded that these changes may contribute to a better environmental adaptation by promoting the digestion and metabolism of food. In another study carried out in frogs, seasonal food and body mass oscillations were significantly correlated with gut microbiota composition suggesting that gut bacteria may change due to dietary pattern variations associated with seasonal environmental changes [28]. Furthermore, a recent study in mice also demonstrated that exposure to regular light/dark cycles or the constant darkness exposure promoted a different gut microbiota profile [29]. In another recently published study with mice housed under different photoperiod conditions, although no significant differences were observed in the overall composition of the gut microbiota, authors were able to extract amplicon sequence variants that were predictive of photoperiod conditions with >91% accuracy [30]. Hence, these studies identify the photoperiod as an important factor which can modulate the gut microbiota composition. However, all of them were done in healthy animals and, therefore, further studies are needed to elucidate the relationship between photoperiod, gut microbiota and diet and its impact on metabolic diseases such as obesity.

Therefore, the aim of the current study was to investigate the effects of photoperiods on fecal microbiota and its impact on body weight gain and different fat depots in healthy and cafeteria-induced obese Fisher 344 rats.

## 2. Materials and Methods

### 2.1. Animals

**Figure 1:**
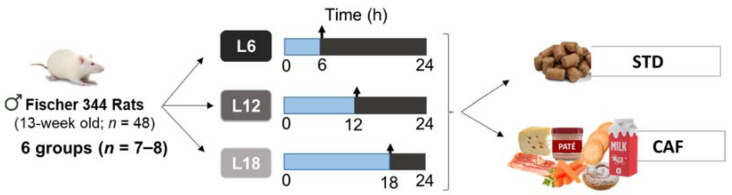
Animal experimental design. 13-week-old male STD- or CAF-fed Fischer 344 rats were pair-housed under three different photoperiods (6, 12 or 18 h of light per day) for 9 weeks. (*n* = 7–8). ♂: represents male sex; L6: short photoperiod (6 h light/18 h dark); L12: standard photoperiod (12 h light/12 h dark); L18: long photoperiod (18 h light/6 h dark); STD: standard chow diet; CAF: cafeteria diet.

Forty-eight 13-week-old male Fisher 344 rats from Janvier Laboratories (Le Genest-Saint-Isle, France) were housed in pairs at standard conditions (22 °C, 65% relative humidity and 12:12 h light/dark cycle). After one week of adaptation to the facility with free access to food and water, animals were weighed and randomly distributed under specific light-dark cycles to simulate three specific photoperiods: short photoperiod (L6, 6 h light/18 h darkness), standard photoperiod (L12, 12 h light/12 h darkness), or long photoperiod (L18, 18 h light/6 h darkness). In each photoperiod, rats were fed either a standard chow diet (STD) (72% carbohydrate, 8% lipid, and 19% protein; Safe-A04c, Scientific Animal Food and Engineering, Barcelona, Spain) or a cafeteria diet (CAF) composed of highly palatable and energy-dense human foods (58% CH, 31% lipid, and 11% protein) for 9 weeks (6 groups, *n* = 7–8 per group) (Figure 1). CAF diet was freshly prepared every day as previously described [31]. Body weight and food intake were recorded weekly during the whole experimental procedure.

Animals were sacrificed by decapitation. Fecal samples were freshly collected from the colon and immediately snap-frozen until further microbiota analysis. The cecum as well as white adipose tissue depots, including mesenteric (mWAT), retroperitoneal (RWAT), inguinal (iWAT), epididymal (eWAT) and subcutaneous, were collected, weighed and immediately frozen in liquid nitrogen. The visceral mass was calculated as the sum of visceral adipose tissue depots (mWAT, RWAT and eWAT). Total body fat mass was measured as the sum of the visceral fat and subcutaneous fat (iWAT and subcutaneous). The adiposity index was expressed as total body fat mass/final body weight. All the samples were stored at −80 °C until further analyses. The Animal Ethics Committee of the Rovira i Virgili University (Tarragona, Spain) and the Generalitat de Catalunya approved all the procedures (number reference 9495) in accordance with the EU Directive 2010/63/EU for animal experiments.

### 2.2. Physical Activity Measurements

Physical activity was evaluated using OxyletProTM system (Panlab, Barcelona, Spain). The measurements were performed at weeks 8 and 9 of the study. Animals were transferred to a standard rodent home cage (Oxylet LE 405 gas analyzer, Panlab) to ensure a contained sample environment. Rats were maintained at 22 °C under the different light/dark cycle conditions, according to the photoperiod, with free access to food and water. The cages were placed on a platform with strain weight transducers to register movements. Data were collected and analyzed. 

### 2.3. 16S rRNA Analysis

DNA from fecal samples was isolated using QiAamp Fast DNA Stool mini kit (Qiagen Inc., Hilden, Germany) and stored at −20 °C until further analysis. The 16S ribosomal RNA sequencing was carried out using an Ion S5 system (Life Technologies, Carlsbad, CA, USA) as described previously [32]. Briefly, V3 and V4 regions were amplified using the following primer pairs: 341F-532R (5′-CCTACGGGRSGCAGCAG-3′; 5′-ATTACCGCGGCTGCT-3′) and 15F-806R (5′-GTGCCAGCMGCCGCGGTAA-3′; 5′-GGACTACHVGGGTWTCTAAT-3′). Specific Ion Torrent compatible adapters and a barcode sequence were added in order to sequence several samples simultaneously. Amplicons were visualized by electrophoresis (2% agarose) and DNA purification was performed with NucleoSpin (Macherey-Nagel, Berlin, Germany). Quality, length, and concentration of the libraries were analyzed using an Agilent 2100 Bioanalyzer (Agilent Technologies, CA, USA). Individual libraries (40 pM) were combined in equimolar amounts in groups of 38 samples. Template preparation and analysis was carried out in an Ion 510 & 520 & Ion 530 Kit-Chef (A34019, Life Technologies, Carlsbad, CA, USA) according to the manufacturer’s instructions. Samples were loaded on a 530 chip (Ion 530TM Chip Kit–4 Reactions) and sequenced using the Ion S5 system (Life Technologies, Carlsbad, CA, USA). Low-quality reads (phred quality score <17) and polyclonal sequences were removed by filtering with the PGM software resulting in a total of 63,212,452 reads. Final sequences were further analyzed by QIIME (quantitative insights into microbial ecology) and GreenGenes database. 

### 2.4. Statistical Analysis

Statistical analysis was performed using SPSS software (IBM SPSS statistics 25, Armonk, NY, USA). In the case of body weight gain, food intake, activity and fats depots data, normality as well as homogeneity of variance were tested by Shapiro–Wilk and Levene test, respectively. Body weight gain over time was analyzed using repeated-measured ANOVA followed by LSD post hoc test at each individual time point. AUC of body weight gain, food intake, activity and fat parameters were analyzed by two-way ANOVA followed by LSD post hoc test. Data were represented as mean ± standard deviation (SD) using Graphpad Prism (v.8.0; Graphapad software Inc., San Diego, CA, USA).

MicrobiomeAnalyst web-based tool [32,33] was used for fecal microbiota analysis. Relative abundance data was filtered (minimum count: 2; prevalence in sample: 10%) in order to exclude low abundance data or those appearing in only one sample. After data filtering, the number of features left was 35,759. Chao1 index and Kruskal–Wallis test were used to calculate and to elucidate alpha diversity differences between groups. Beta diversity was calculated based on Bray–Curtis distances and analyzed by permutational multivariate analysis of variance (PERMANOVA). Differences in relative abundance of specific bacteria taxa were analyzed using either Mann–Whitney (if comparing two groups) or Kruskal–Wallis test followed by Dunn’s multiple comparison with Bonferroni adjustment of *p* values.

Spearman’s rank-order correlation analysis between fecal microbiota at different taxonomic levels with body weight gain and fat parameters was carried out using Python script as previously described [31]. The FDR (false discovery rate) control for *p*-value correction in multiple comparisons was applied. The script was developed using PyCharm software (v.2018.2.4, JetBrains s.r.o., Prague, Czech Republic) and Python version 3.7.7. 

Statistical significances were depicted as follows: *indicating diet effect *p* < 0.05, and ab letters indicating photoperiod effect *p* < 0.05.

## 3. Results

### 3.1. Photoperiod Effect on Body Weight

**Figure 2:**
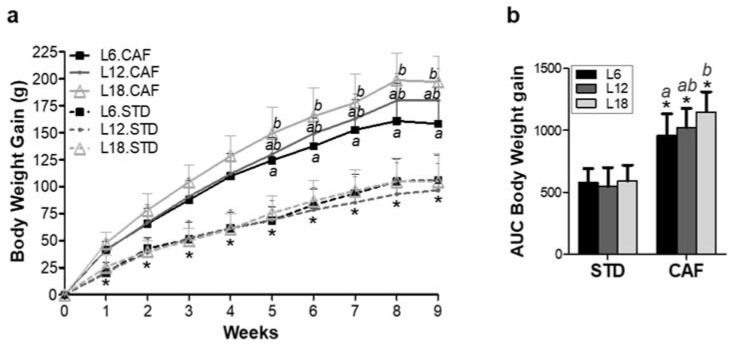
Effects of photoperiods on body weight gain in STD- and CAF-fed rats. (**a**) Body weight gain under short (L6), standard (L12) and long (L18) photoperiods across the 9 weeks of the experiment. * indicates significant CAF effect and *a,b* letters indicate significant CAF and photoperiod effects respectively, analyzed by repeated measures ANOVA followed by LSD post hoc test (*p* < 0.05). (**b**) Area under the curve (AUC) of body weight gain. * indicates significant CAF effect and *a,b* letters indicate photoperiod effect, analyzed by 2-way ANOVA followed by LSD post hoc test (*p* < 0.05). Data are plotted as the mean ± SD (*n* = 7–8). L6: short photoperiod (6 h light/18 h dark); L12: standard photoperiod (12 h light/12 h dark); L18: long photoperiod (18 h light/6 h dark); STD: standard chow diet; CAF: cafeteria diet.

CAF-fed rats showed a significant increased body weight gain (*p* < 0.001) and corresponding AUC compared to STD-fed rats under the three different photoperiod conditions across the 9 weeks of the experiment (Figure 2a,b).

Exposure to different photoperiods did not affect body weight gain in STD-fed rats (Figure 2). In contrast, CAF-fed rats exposed to the long photoperiod (L18) showed higher body weight gain during the last 5 weeks of the experiment (weeks 5–9) and a significantly higher corresponding area under the curve (AUC) when compared to rats exposed to the short photoperiod (L6) (*p* < 0.05) (Figure 2). These changes in body weight gain were not associated either with higher food intake (Figure S1a–f) or with lower activity in rats housed under L18 conditions (Figure S1g,h).

### 3.2. Photoperiods Affect Fecal Microbiota Composition: Higher Impact on Cafeteria Diet-Fed Rats

**Figure 3:**
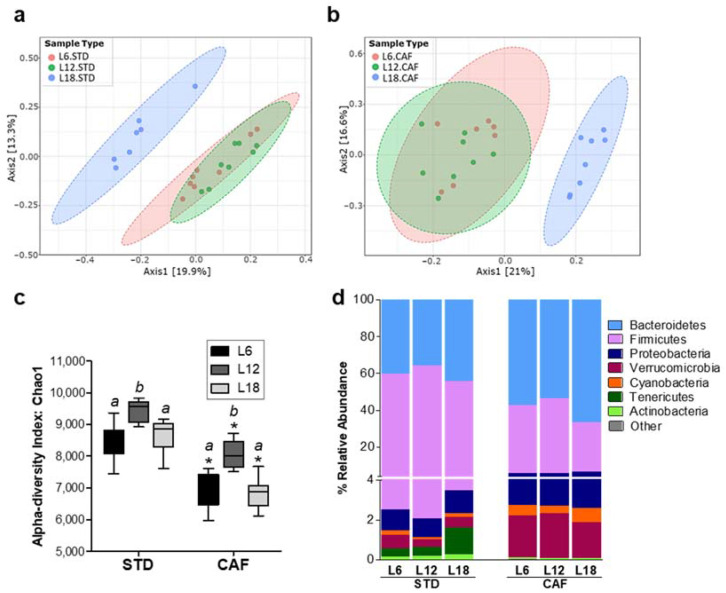
Effect of photoperiods (Ph) on both fecal microbial diversity and bacteria phyla relative abundance. Principal coordinates analysis (PCoA) 2D plot (PERMANOVA, *p* < 0.001) of fecal microbiota beta diversity based on Bray–Curtis distances in (**a**) STD- and in (**b**) CAF-fed rats; (**c**) alpha diversity calculated by chao-1 index in STD- and CAF-fed rats under the three different Ph conditions. Data are plotted as box and whiskers (median with interquartile ranges). * Indicates significant diet effect between STD and CAF-fed rats under same photoperiod conditions, analyzed by U-Mann–Whitney (*p* < 0.05); *a,b* letters indicate significant photoperiod effect analyzed by Kruskal–Wallis test followed by Bonferroni correction for multiple comparisons (*p* < 0.016); (**d**) relative abundance of different bacteria taxa at phylum level. (*n* = 7–8). L6: short photoperiod (6 h light/18 h dark); L12: standard photoperiod (12 h light/12 h dark); L18: long photoperiod (18 h light/6 h dark); STD: standard chow diet; CAF: cafeteria diet.

PERMANOVA analysis of fecal microbiota beta diversity revealed a significant CAF effect under each photoperiod condition (Figure S2). In addition, a significant photoperiod effect in both STD- (*p* < 0.001) and CAF-fed (*p* < 0.001) rats under L18 conditions was found (Figure 3a,b). Interestingly, the CAF effect on rats housed under L18 conditions was stronger than in both L6 and L12. Thus, samples were grouped according to diet type along the PC1 axis. (Figure S2c).

CAF feeding significantly reduced fecal microbiota diversity independently of photoperiod exposure (*p* < 0.01) (Figure 3c). Remarkably, fecal microbiota alpha diversity also showed an interesting photoperiod effect. Both STD- and CAF-fed rats under L12 showed a significant higher alpha diversity than rats under L6 and L18 (*p* < 0.05) (Figure 3c).

The relative abundance at phylum level was analyzed to evaluate photoperiod and CAF effects on fecal microbiota composition. A significant effect of CAF feeding on phyla relative abundance was observed independently of photoperiod exposure (Figure 3d, Table S1). Thus, CAF feeding led to a significant increase in Bacteroidetes, Proteobacteria, Verrucromicrobia and Cyanobacteria and a decrease in Firmicutes and Tenericutes (*p* < 0.05). Moreover, the Firmicutes and Bacteroidetes alteration by CAF feeding caused a significant decrease in the Firmicutes to Bacteroidetes ratio (*p* < 0.016) (Table S1).

Regarding the photoperiod effect, STD-fed rats did not show a photoperiod effect on fecal bacteria relative abundance at phylum level, whereas CAF-fed rats showed a trend towards decreased Firmicutes (*p* = 0.07) and increased Bacteroidetes (*p* = 0.08) relative abundance levels under L18 compared to both L6 and L12 (Figure 3d). Besides this trend effect under L18, no photoperiod effects were observed on the Firmicutes to Bacteroidetes ratio (F/B ratio) (Table S1).

**Figure 4:**
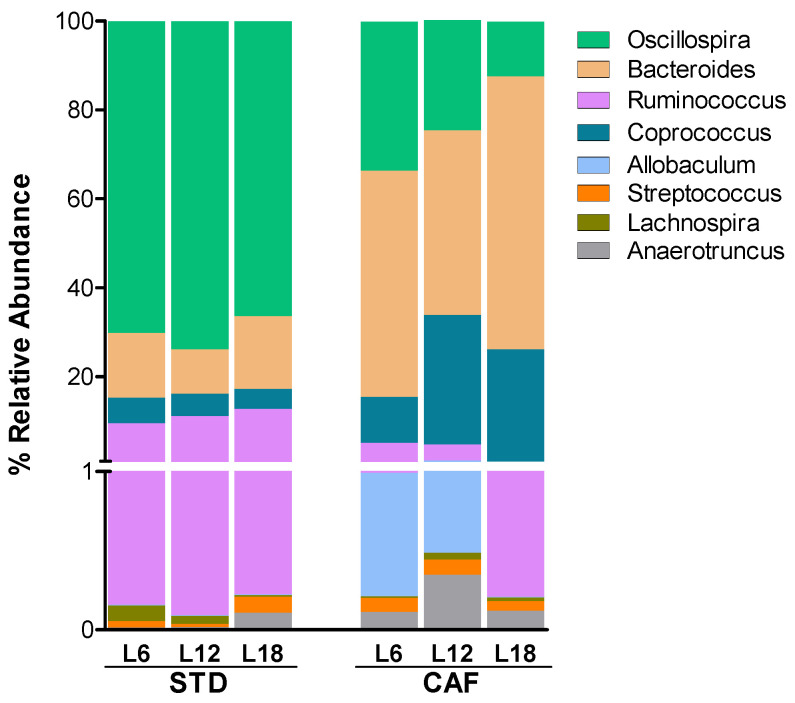
Relative abundance at genus level of the most abundant genera significantly altered by photoperiods. Stacked bar plots showing the relative abundance of each taxa at genus level. (*n* = 7–8). L6: short photoperiod (6 h light/18 h dark); L12: standard photoperiod (12 h light/12 h dark); L18: long photoperiod (18 h light/6 h dark); STD: standard chow diet; CAF: cafeteria diet.

When looking at genera level, several of the bacteria genera relative abundances were affected by CAF feeding (Figure 4; Table S2). Thus, changes in genera belonging to Firmicutes and Bacteroidetes phyla were observed in CAF-fed rats while changes in less abundant genera (relative abundance <0.1%) belonging to Actinobacteria, Bacteroidetes, Firmicutes and Proteobacteria phyla were observed in STD-fed rats. 

Furthermore, photoperiod housing conditions also affected gut microbiota composition at this taxonomical level, mainly in CAF-fed rats (Figure 4; Table S2). Thus, it is worth highlighting some of the most abundant genera which altered significantly among photoperiods. Bacteroides, one of the most abundant genera that was increased by CAF feeding, increased in rats housed under L18 conditions. Oscillospira and Ruminococcus, which were significantly decreased by CAF feeding, showed significantly lower levels in rats housed under L18 conditions compared to those housed under L6. Other bacteria genera such as Coprococcus and Allobaculum, which were increased by CAF feeding, were also altered by photoperiod (Figure 4).

### 3.3. Correlations between Fecal Microbiota Taxa, Body Weight Gain and Fat Parameters

**Figure 5:**
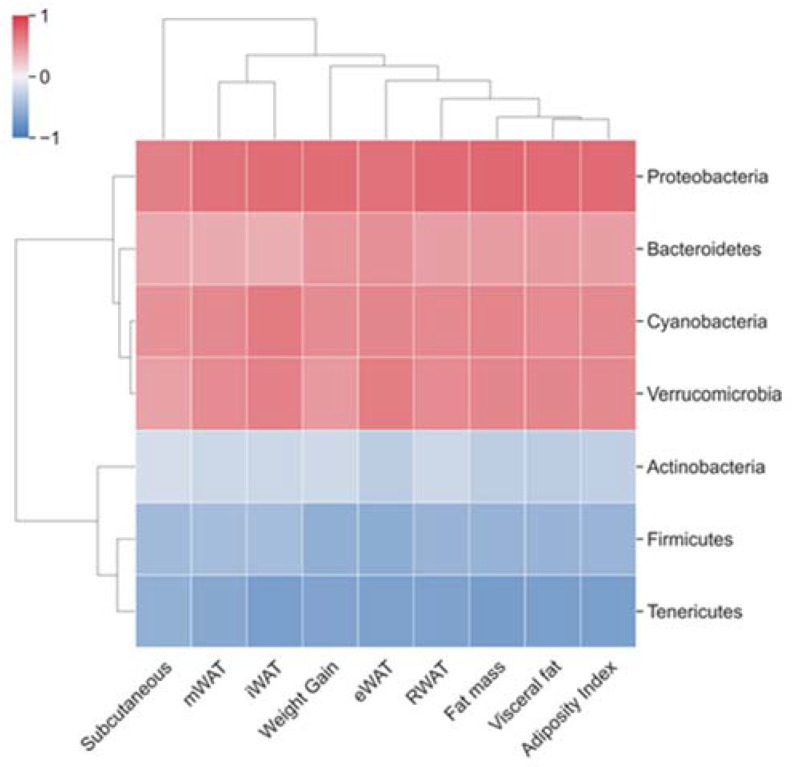
Correlations between fecal microbiota and body weight gain and fat parameters analyzed by Spearman’s rank correlation coefficient (rho) at phylum level. Heat map with hierarchical clustering based on correlation coefficient between bacteria and biometric parameters at phylum level. Positive and negative correlations are represented in red and blue respectively. The higher the color intensity the higher the degree of correlation.

Bacteria taxa significantly altered by CAF or photoperiod conditions were selected in order to investigate if they correlated with body weight and fat parameters (fat depots accumulation, fat mass, visceral mass and adiposity index; Figure S3). Several correlations were observed (Table S3) and two main clusters were identified at phylum level. The first cluster involved Proteobacteria, Bacteroidetes, Cyanobacteria and Verrucromicrobia phyla showing positive correlations with the different fat parameters. The second cluster included Actinobacteria, Firmicutes and Tenericutes phyla showing negative correlations with these parameters (Figure 5). Proteobacteria and Firmicutes, two of the most abundance phyla, showed the highest number of strong to moderate significant correlations with iWAT, RWAT, visceral fat, fat mass and adiposity index (rho < 0.5/rho < −0.5, *p* < 0.05, FDR < 0.05) (Table S3). The analysis at family level showed strong and moderate positive correlations of *Lachnospiraceae*, *Bacteroidaceae*, *Streptococcaceae* and *Verrucomicrobiaceae* with the different analyzed parameters (rho = 0.7–0.5, *p* < 0.05, FDR < 0.05), while, *Clostridiaceae* and *Ruminococcaceae* presented strong negative correlations (rho = −0.7–−0.5, *p* < 0.05, FDR < 0.05) (Table S3).

**Figure 6:**
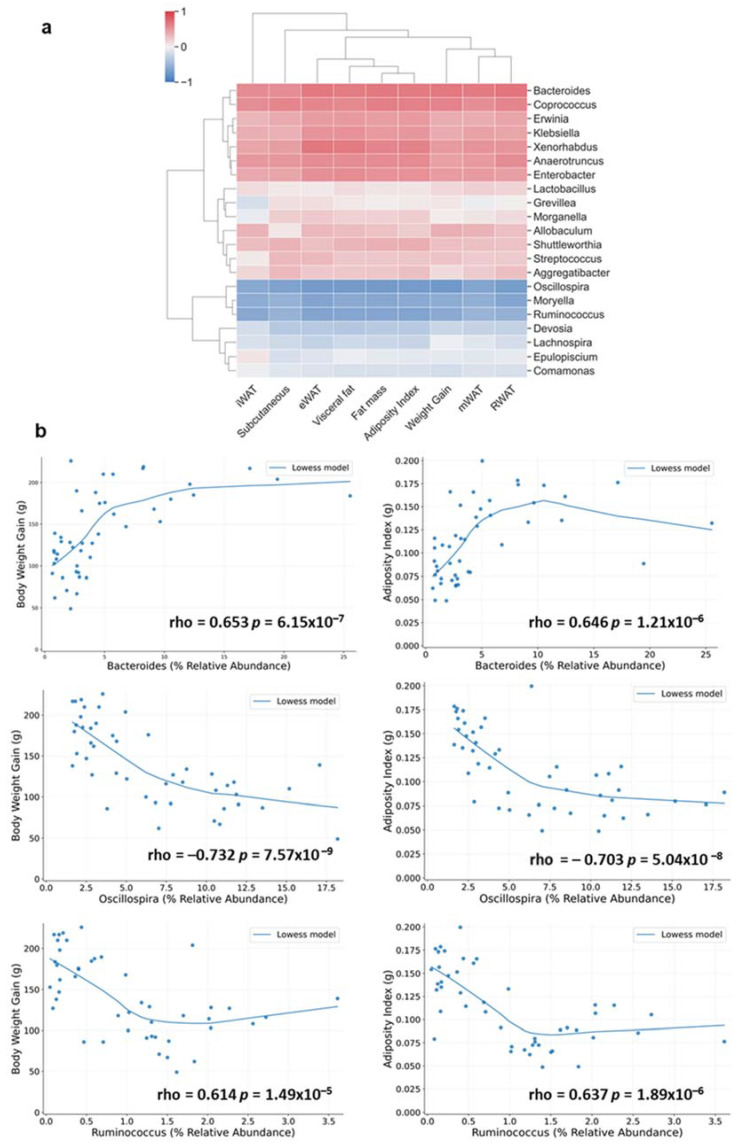
Correlations between fecal microbiota and fat parameters analyzed by Spearman’s rank correlation coefficient (rho) at genus level. (**a**) Heat map with hierarchical clustering based on correlation coefficient between bacteria and fat parameters at genus level. Positive and negative correlations are represented in red and blue, respectively. The higher the color intensity the higher the degree of correlation. (**b**) Locally weighted linear regression (Lowess model) analysis of the strongest observed correlation in several bacteria genera affected by photoperiod.

Since the assessment of these results revealed significant correlations between the relative abundance of different bacteria taxa and the different fat parameters, we further investigated these associations at genera level, focusing only on bacteria significantly altered by photoperiod conditions. Two clear clusters were identified: a first remarkable cluster positively correlated with the fat parameters, involving principally bacteria belonging to the Firmicutes, Bacteroidetes and Proteobacteria phyla, and a second cluster negatively correlated with the different fat parameters, involving mostly bacteria belonging to the Firmicutes phyla (Figure 6a). It is worth highlighting the strongest correlations observed in both clusters. Thus, *Bacteroides* and *Coprococcus* genera (belonging to Bacteroidetes and Firmicutes phyla respectively) showed a higher positive correlation with mWAT, RWAT, fat mass, visceral fat, adiposity index and body weight gain (rho = 0.67–0.6, *p* < 0.001, FDR < 0.05) (Figure 6b). On the other hand, in the second cluster, strong negative correlations with the different fat depots and body weight gain were observed for *Oscillospira* and *Ruminococcus* genera (rho = 0.6–0.7, *p* < 0.001, FDR < 0.05) (Figure 6b).

## 4. Discussion

In the latest years, several studies have demonstrated that gut bacteria significantly affect host metabolism and physiology [1]. This has led to an increasing interest in understanding how gut microbiota composition is modulated. Dietary pattern is among the main factors that shape these gut microbes [34], but other environmental and intrinsic factors such as antibiotic intake [35], age [36], gender [37], physical activity [38] or stress [39] may be also involved. In addition to these factors, the exposure to different light cycles has recently been demonstrated to impact gut microbiota composition [21,29]. This is important as changes in gut microbiota composition may lead to different metabolic and physiologic responses, contributing to the adaptation to changes in environmental conditions associated to the different seasons. However, the relationship between seasonal rhythms and gut microbiota and its impact on the host physiology is still poorly understood. Hence, as mentioned earlier, only a few studies have focused on investigating seasonal variations of gut bacteria. Moreover, these studies have used non-obese animals and therefore the effects of seasonal variations under an obesogenic context has not been sufficiently investigated yet [24,25,27,40]. Therefore, we investigated the effect of different photoperiods on gut microbiota composition in both healthy and obese rats and how those changes correlated with parameters related to obesity development such as body weight gain and fat depots accumulation.

Obesity was induced by cafeteria diet feeding. This diet is a well-established model to induce obesity and other pathologies related to the metabolic syndrome and consists of highly palatable foods that lead to high caloric intake with poor nutritional value contributing to the development of different disorders such as insulin resistance, metabolic disruption and alterations of the gut microbiota composition [41,42]. Indeed, CAF-fed rats showed higher body weight gain, higher adiposity accumulation and gut microbiota dysbiosis compared to STD-fed rats. Additionally, obesity has been widely related with a reduction of alpha microbial diversity [43] and an increase of the Firmicutes to Bacteroidetes ratio in obese humans and animals [44]. In this context, CAF-fed rats showed lower alpha diversity but the Firmicutes/Bacteroidetes ratio was decreased due to the increase of Bacteroidetes and the reduction of Firmicutes relative abundance. However, this is in accordance with other studies using this type of cafeteria diet [4,45]. This discrepancy regarding Firmicutes/Bacteroidetes ratio with other high fat diets induced obesity models may be promoted by differences in the type of fat present in the diets, mainly lard and milk-derived fat-based diets [46]. Thus, the conflicting effects of CAF and other high fat diets on the Firmicutes/Bacteroidetes ratio may be explained by higher consumption of milk fat in CAF and higher intake of lard in other high fat diets. Indeed, clinical studies have also demonstrated that increased Firmicutes/Bacteroidetes ratio is not always related to obesity [47]. Hence, the association of this ratio with obesity should be considered carefully. Moreover, it is worth highlighting that CAF feeding did also significantly alter other phyla such as Proteobacteria and Verrucromicrobia, and other bacteria relative abundances at different taxonomic levels such as *Clostridiaceae*, *Lachnospiraceae* and *Prevotellaceae* at family level and *Bacteroides*, *Oscillospira*, *Ruminococcus* and *Akkermansia* at genus level, which have been related with obesity and metabolic disorders [48]. 

Different photoperiod conditions were used to simulate seasonal rhythms. Thus, the short photoperiod conditions emulated the hours of light in short days typical of the winter season while the long photoperiod conditions simulated the long days typical of the summer season. Interestingly, the photoperiod conditions significantly affected the overall fecal microbiota profile, and these changes were associated with differences in body weight gain and fat content. These results are in accordance with previous studies in Siberian hamsters, which showed variations in gut microbiota composition caused by different photoperiod conditions [21,23]. In particular, we observed a decreased alpha microbial diversity under L6 and L18 compared to L12 in both STD- and CAF-fed rats. This is in accordance with a previous studies that found that alpha diversity of fecal microbiota was significantly decreased in mice under 24 h light conditions compared to those under normal 12-h LD cycles, suggesting that light cycles help to maintain a higher variety of gut microbiota [49]. In addition, rats housed under L18 conditions showed a significant different overall gut microbiota composition as elucidated by beta diversity analysis in both the STD- and CAF-fed diet. Interestingly, CAF-fed rats housed under this photoperiod condition also showed higher body weight gain and fat content. Remarkably, the increase in these parameters was not due to a change either in diet or in activity. This is common in mammals which are able to adapt to changes in the environment driven by changes in the light and dark cycle during the different seasons [50]. Hence, one specific trait of seasonal manifestation in mammals is a more efficient pattern of energy harvesting, expenditure and storage during the reproductive part of the year, which usually happens under the long photoperiod. In contrast, energy exploitation is scarce during the short photoperiod, which usually corresponds to the unproductive season [51,52]. Thus, it seems that the enhanced masses may be due to differences in the ability of the rats to harvest energy from the consumed food, being more efficient under L18 conditions. In addition, the gut microbiota profile from obese animals has been shown to have a higher capacity to harvest energy from the diet due to an increased glucose absorption and fatty acid absorption and production [53,54]. CAF-fed rats housed under L18 showed higher abundance of *Bacteroidetes* and lower abundance of *Firmicutes*. Indeed, *Bacteroidetes* was positively correlated with the body composition while *Firmicutes* was negatively correlated with these parameters. Interestingly, both phyla are often involved in carbohydrate metabolism [55,56]. The products of carbohydrate fermentation provide the host with energy, supporting the idea that these phyla are associated with an obesity susceptibility in the host [57]. In CAF-fed rats, most of the bacteria genera altered by photoperiod belonged to Firmicutes and Bacteroidetes phyla. Interestingly, two of the most abundant genera, *Oscillospira* and *Ruminococcus* were decreased in CAF rats under L18 and correlated negatively with the biometric parameters. These genera have been shown to be decreased in obese subjects and are known as potential butyrate producers [58,59]. This short chain fatty acid has been demonstrated to exert beneficial effects against obesity by increasing energy expenditure and lipid oxidation [60]. In addition, *Bacteroides* genera, prominent among obese individuals, was increased in this group and correlated positively with body composition. Therefore, these results revealed a relationship between gut microbiota and body weight gain and fat depots that might be driven by photoperiod conditions. 

Finally, it is remarkable that STD-fed rats also showed a photoperiod effect on the fecal microbiota composition. However, these changes were observed in genera with a low relative abundance (<0.1%). In addition, these changes were not associated with differences in the body weight gain and fat depots, which were not affected by photoperiod in STD-fed rats. These findings were in agreement with previous work by our laboratory [20,61], but in contrast with other studies that showed significant changes in body weight gain and fat depot accumulation after a chronic exposure to different photoperiods in STD-fed rats [62,63]. This absence of variation in these parameters may be due to a potential adaptative response to chronic short photoperiod exposition, ensuring survival and avoiding reproductive suppression [64]. Thus, these results could mean that there is an interaction between photoperiod, diet and gut microbiota, obese-induced diet rats being more susceptible to photoperiod. 

## 5. Conclusions

In conclusion, the current study suggests an interaction between photoperiod and gut microbiota being linked to metabolic disorders such as obesity. This interaction, which affects the body composition, may also affect physiological responses. Therefore, our research can set the basis to understand the potential benefits of microbiota-targeted therapies and to continue the study of the mechanisms regulating seasonal shifts associated with the development of metabolic diseases such as obesity. 

## Data Availability

The data presented in this study are available, on request from authors, in Zenodo repository at doi:10.5281/zenodo.5785702.

## Supplementary Materials

The following supporting information can be downloaded at: https://www.mdpi.com/article/10.3390/nu14030722/s1, Figure S1: Food intake and physical activity, Figure S2: Effect of diet on the beta-diversity in STD and CAF groups, Figure S3: Effects of photoperiod on white adipose tissue depots and cecum weight, Table S1: Relative abundance at phylum level of STD- and CAF-fed rats under the three different photoperiod conditions (L6, L12 and L18), Table S2: Significant photoperiod and diet effect at genus level, Table S3: Significant Spearman’s correlations between body weight gain and fat parameters with the relative abundance bacteria at different taxonomic levels.

## References

[1] Patterson E., Ryan P.M., Cryan J.F., Dinan T.G., Ross R.P., Fitzgerald G.F., Stanton C. (2016). Gut microbiota, obesity and diabetes. Postgrad. Med. J..

[2] Torres-Fuentes C., Schellekens H., Dinan T.G., Cryan J.F. (2017). The microbiota–gut–brain axis in obesity. Lancet Gastroenterol. Hepatol..

[3] Sandhu K.V., Sherwin E., Schellekens H., Stanton C., Dinan T., Cryan J.F. (2017). Feeding the microbiota-gut-brain axis: Diet, microbiome, and neuropsychiatry. Transl. Res..

[4] Del Chierico F., Vernocchi P., Dallapiccola B., Putignani L. (2014). Mediterranean diet and health: Food effects on gut microbiota and disease control. Int. J. Mol. Sci..

[5] Shi Z. (2019). Gut microbiota: An important link between western diet and chronic diseases. Nutrients.

[6] Del Bas J.M., Guirro M., Boqué N., Cereto A., Ras R., Crescenti A., Caimari A., Canela N., Arola L. (2018). Alterations in gut microbiota associated with a cafeteria diet and the physiological consequences in the host. Int. J. Obes..

[7] Nobs S.P., Tuganbaev T., Elinav E. (2019). Microbiome diurnal rhythmicity and its impact on host physiology and disease risk. EMBO Rep..

[8] Thaiss C.A., Levy M., Korem T., Dohnalová L., Shapiro H., Jaitin D.A., David E., Winter D., Gury-BenAri M., Tatirovsky E. (2016). Microbiota Diurnal Rhythmicity Programs Host Transcriptome Oscillations. Cell.

[9] Claustrat B., Leston J. (2015). Melatonin: Physiological effects in humans. Neurochirurgie.

[10] Johnston J.D., Ordovás J.M., Scheer F.A., Turek F.W. (2016). Circadian rhythms, metabolism, and chrononutrition in rodents and humans. Adv. Nutr..

[11] Refinetti R. (2012). Integration of biological clocks and rhythms. Compr. Physiol..

[12] Rácz B., Dušková M., Stárka L., Hainer V., Kunešová M. (2018). Links between the circadian rhythm, obesity and the microbiome. Physiol. Res..

[13] Onishi K.G., Maneval A.C., Cable E.C., Tuohy M.C., Scasny A.J., Sterina E., Love J.A., Riggle J.P., Malamut L.K., Mukerji A. (2019). Circadian and circannual timescales interact to generate seasonal changes in immune function. Brain Behav. Immun..

[14] Murphy B.A. (2019). Circadian and Circannual Regulation in the Horse: Internal Timing in an Elite Athlete. J. Equine Veter. Sci..

[15] Tackenberg M.C., McMahon D.G. (2018). Photoperiodic Programming of the SCN and Its Role in Photoperiodic Output. Neural Plast..

[16] Coomans C., Ramkisoensing A., Meijer J.H. (2015). The suprachiasmatic nuclei as a seasonal clock. Front. Neuroendocr..

[17] Haus E. (2007). Chronobiology in the endocrine system. Adv. Drug Deliv. Rev..

[18] Vitale J.A., Briguglio M., Galentino R., Dell’Osso B., Malgaroli A., Banfi G., Porta M. (2020). Exploring circannual rhythms and chronotype effect in patients with Obsessive-Compulsive Tic Disorder (OCTD): A pilot study. J. Affect. Disord..

[19] Jin J., Yaegashi T., Hashizume T. (2013). Effects of photoperiod on the secretion of growth hormone and prolactin during nighttime in female goats. Anim. Sci. J..

[20] Mariné-Casadó R., Coca C.D., Del Bas J.M., Bladé C., Arola L., Caimari A. (2018). The exposure to different photoperiods strongly modulates the glucose and lipid metabolisms of normoweight fischer 344 rats. Front. Physiol..

[21] Bailey M.T., Walton J.C., Dowd S., Weil Z., Nelson R.J. (2010). Photoperiod modulates gut bacteria composition in male Siberian hamsters (Phodopus sungorus). Brain Behav. Immun..

[22] Davenport E., Mizrahi-Man O., Michelini K., Barreiro L., Ober C., Gilad Y. (2014). Seasonal variation in human gut microbiome composition. PLoS ONE.

[23] Shor E.K., Brown S.P., Freeman D.A. (2020). A novel role for the pineal gland: Regulating seasonal shifts in the gut microbiota of Siberian hamsters. J. Pineal Res..

[24] Fan C., Zhang L., Jia S., Tang X., Fu H., Li W., Liu C., Zhang H., Cheng Q., Zhang Y. (2022). Seasonal variations in the composition and functional profiles of gut microbiota reflect dietary changes in plateau pikas. Integr. Zool..

[25] Huang G., Wang L., Li J., Hou R., Wang M., Wang Z., Qu Q., Zhou W., Nie Y., Hu Y. (2022). Seasonal shift of the gut microbiome synchronizes host peripheral circadian rhythm for physiological adaptation to a low-fat diet in the giant panda. Cell Rep..

[26] Yang X., Yao Y., Zhang X., Zhong J., Gao F., Zhang H., Han Y., Weng Q., Yuan Z. (2021). Seasonal Changes in the Distinct Taxonomy and Function of the Gut Microbiota in the Wild Ground Squirrel (*Spermophilus dauricus*). Animals.

[27] Jiang F., Gao H., Qin W., Song P., Wang H., Zhang J., Liu D., Wang D., Zhang T. (2021). Marked Seasonal Variation in Structure and Function of Gut Microbiota in Forest and Alpine Musk Deer. Front. Microbiol..

[28] Huang C., Liao W. (2021). Seasonal variation in gut microbiota related to diet in *Fejervarya limnocharis*. Animals.

[29] Wu G., Tang W., He Y., Hu J., Gong S., He Z., Wei G., Lv L., Jiang Y., Zhou H. (2018). Light exposure influences the diurnal oscillation of gut microbiota in mice. Biochem. Biophys. Res. Commun..

[30] Oyola M.G., Johnson R.C., Bauman B.M., Frey K.G., Russell A.L., Cho-Clark M., Buban K.N., Bishop-Lilly K.A., Merrell D.S., Handa R.J. (2021). Gut microbiota and metabolic marker alteration following dietary isoflavone-photoperiod interaction. Endocrinol. Diabetes Metab..

[31] Ávila-Román J., Arreaza-Gil V., Cortés-Espinar A.J., Soliz-Rueda J.R., Mulero M., Muguerza B., Arola-Arnal A., Arola L., Torres-Fuentes C. (2021). Impact of gut microbiota on plasma oxylipins profile under healthy and obesogenic conditions. Clin. Nutr..

[32] Dhariwal A., Chong J., Habib S., King I.L., Agellon L.B., Xia J. (2017). MicrobiomeAnalyst: A web-based tool for comprehensive statistical, visual and meta-analysis of microbiome data. Nucleic Acids Res..

[33] Chong J., Liu P., Zhou G., Xia J. (2020). Using MicrobiomeAnalyst for comprehensive statistical, functional, and meta-analysis of microbiome data. Nat. Protoc..

[34] Voreades N., Kozil A., Weir T.L. (2014). Diet and the development of the human intestinal microbiome. Front. Microbiol..

[35] Gibson M.K., Crofts T.S., Dantas G. (2015). Antibiotics and the developing infant gut microbiota and resistome. Curr. Opin. Microbiol..

[36] Coman V., Vodnar D.C. (2020). Gut microbiota and old age: Modulating factors and interventions for healthy longevity. Exp. Gerontol..

[37] Kim Y.S., Unno T., Kim B.-Y., Park M.-S. (2020). Sex Differences in Gut Microbiota. World J. Mens Health.

[38] O’Sullivan O., Cronin O., Clarke S.F., Murphy E.F., Molloy M.G., Shanahan F., Cotter P. (2015). Exercise and the microbiota. Gut Microbes.

[39] Rea K., Dinan T., Cryan J.F. (2016). The microbiome: A key regulator of stress and neuroinflammation. Neurobiol. Stress.

[40] Ren C.C., Sylvia K.E., Munley K.M., Deyoe J.E., Henderson S.G., Vu M.P., Demas G.E. (2020). Photoperiod modulates the gut microbiome and aggressive behavior in Siberian hamsters. J. Exp. Biol..

[41] Macedo I.C., de Freitas J.S., Torres I.L.D.S. (2016). The influence of palatable diets in reward system activation: A mini review. Adv. Pharmacol. Sci..

[42] Guirro M., Costa A., Gual-Grau A., Herrero P., Torrell H., Canela N., Arola L. (2019). Effects from diet-induced gut microbiota dysbiosis and obesity can be ameliorated by fecal microbiota transplantation: A multiomics approach. PLoS ONE.

[43] Le Chatelier E., Nielsen T., Qin J., Prifti E., Hildebrand F., Falony G., Almeida M., Arumugam M., Batto J.-M., Kennedy S. (2013). Richness of human gut microbiome correlates with metabolic markers. Nature.

[44] Zhang C., Zhang M., Pang X., Zhao Y., Wang L., Zhao L. (2012). Structural resilience of the gut microbiota in adult mice under high-fat dietary perturbations. ISME J..

[45] Gual-Grau A., Guirro M., Mayneris-Perxachs J., Arola L., Boqué N. (2019). Impact of different hypercaloric diets on obesity features in rats: A metagenomics and metabolomics integrative approach. J. Nutr. Biochem..

[46] Devkota S., Wang Y., Musch M.W., Leone V., Fehlner-Peach H., Nadimpalli A., Antonopoulos D.A., Jabri B., Chang E.B. (2012). Dietary-fat-induced taurocholic acid promotes pathobiont expansion and colitis in Il10-/- mice. Nature.

[47] Magne F., Gotteland M., Gauthier L., Zazueta A., Pesoa S., Navarrete P., Balamurugan R. (2020). The firmicutes/bacteroidetes ratio: A relevant marker of gut dysbiosis in obese patients?. Nutrients.

[48] Crovesy L., Masterson D., Rosado E.L. (2020). Profile of the gut microbiota of adults with obesity: A systematic review. Eur. J. Clin. Nutr..

[49] Deaver J.A., Eum S.Y., Toborek M. (2018). Circadian disruption changes gut microbiome taxa and functional gene composition. Front. Microbiol..

[50] Goldman B.D. (2001). Mammalian photoperiodic system: Formal properties and neuroendocrine mechanisms of photoperiodic time measurement. J. Biol. Rhythm..

[51] Varpe O.H. (2017). Life History Adaptations to Seasonality. Integrative and Comparative Biology.

[52] Hut R.A., Beersma D.G.M. (2011). Evolution of time-keeping mechanisms: Early emergence and adaptation to photoperiod. Philos. Trans. R. Soc. B Biol. Sci..

[53] Jumpertz R., Le D.S., Turnbaugh P.J., Trinidad C., Bogardus C., Gordon J.I., Krakoff J. (2011). Energy-balance studies reveal associations between gut microbes, caloric load, and nutrient absorption in humans. Am. J. Clin. Nutr..

[54] Bäckhed F., Ding H., Wang T., Hooper L.V., Koh G.Y., Nagy A., Semenkovich C.F., Gordon J.I. (2004). The gut microbiota as an environmental factor that regulates fat storage. Proc. Natl. Acad. Sci. USA.

[55] Ramakrishna B. (2013). Role of the gut microbiota in human nutrition and metabolism. J. Gastroenterol. Hepatol..

[56] Samuel B.S., Shaito A., Motoike T., Rey F.E., Backhed F., Manchester J.K., Hammer R.E., Williams S.C., Crowley J., Yanagisawa M. (2008). Effects of the gut microbiota on host adiposity are modulated by the short-chain fatty-acid binding G protein-coupled receptor, Gpr41. Proc. Natl. Acad. Sci. USA.

[57] Ottman N., Smidt H., De Vos W.M., Belzer C. (2012). The function of our microbiota: Who is out there and what do they do?. Front. Cell. Infect. Microbiol..

[58] Raman M., Ahmed I., Gillevet P.M., Probert C.S., Ratcliffe N.M., Smith S., Greenwood R., Sikaroodi M., Lam V., Crotty P. (2013). Fecal microbiome and volatile organic compound metabolome in obese humans with nonalcoholic fatty liver disease. Clin. Gastroenterol. Hepatol..

[59] Zarrinpar A., Chaix A., Yooseph S., Panda S. (2014). Diet and feeding pattern affect the diurnal dynamics of the gut microbiome. Cell Metab..

[60] Gao Z., Yin J., Zhang J., Ward R.E., Martin R.J., Lefevre M., Cefalu W.T., Ye J. (2009). Butyrate improves insulin sensitivity and increases energy expenditure in mice. Diabetes.

[61] Mariné-Casadó R., Domenech-Coca C., Del Bas J.M., Bladé C., Arola L., Caimari A. (2018). Intake of an Obesogenic Cafeteria Diet Affects Body Weight, Feeding Behavior, and Glucose and Lipid Metabolism in a Photoperiod-Dependent Manner in F344 Rats. Front. Physiol..

[62] Shoemaker M.B., Heideman P.D. (2002). Reduced body mass, food intake, and testis size in response to short photoperiod in adult F344 rats. BMC Physiol..

[63] Tavolaro F.M., Thomson L.M., Ross A., Morgan P., Helfer G. (2015). Photoperiodic Effects on Seasonal Physiology, Reproductive Status and Hypothalamic Gene Expression in Young Male F344 Rats. J. Neuroendocr..

[64] Heideman P.D., Sylvester C.J. (1997). Reproductive Photoresponsiveness in Unmanipulated Male Fischer 344 Laboratory Rats1. Biol. Reprod..

