# Supplementary material for: Gut Seasons: Photoperiod Effects on Fecal Microbiota in Healthy and Cafeteria-Induced Obese Fisher 344 Rats
Source: Nutrients. 2022 Feb 8;14(3):722. doi: 10.3390/nu14030722 (PMC8839759; doi:10.3390/nu14030722)
Supplement: Supplementary file 1 [file nutrients-14-00722-s001.zip › nutrients-1575878-supplementary.pdf]

Gut seasons: Photoperiod effects on faecal microbiota in healthy and cafeteria-induced obese Fisher 344 Rats.

Verónica Arreaza-Gil, Iván Escobar-Martínez, Manuel Suárez, Francisca Isabel Bravo, Begoña Muguerza, Anna Arola-Arnal*, Cristina Torres-Fuentes

Nutrigenomics Research Group, Departament de Bioquímica i Biotecnologia, Universitat Rovira i Virgili, 43007 Tarragona, Spain; veronica.arreaza@urv.cat (V. A-G); ivan.escobar@urv.cat (I. E-M); manuel.suarez@urv.cat (M.S); franciscaisabel@urv.cat (F.I.B); begona.muguerza@urv.cat (B.M.); cristina.torres@urv.cat (C. T-F).
* Correspondence: anna.arola@urv.cat

Supplementary Material

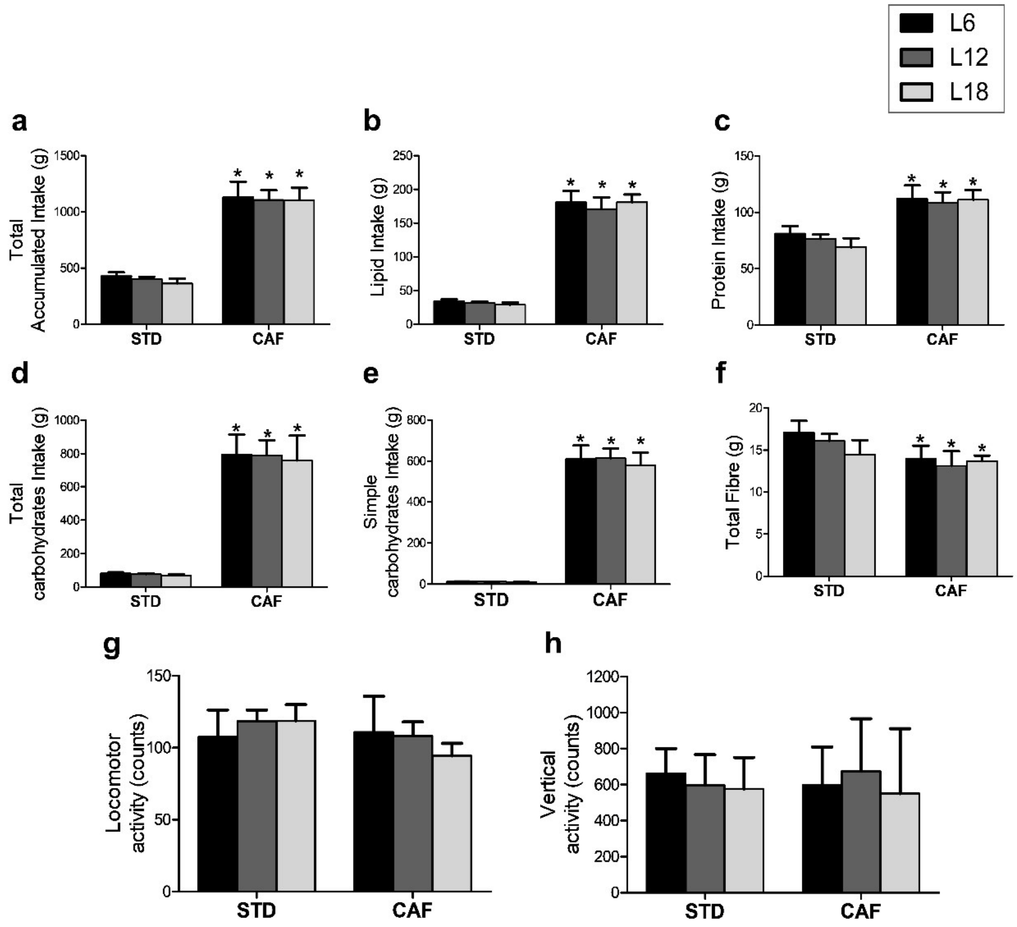

Figure S1. Food intake and Physical activity. (**a** to **f**): Accumulate food intake (**a**) and total intake derived from each macronutrient within each dietary groups, showing lipid (**b**), protein (**c**), complex carbohydrates (**d**), simple carbohydrates (**e**) and fibre (**f**) consumed by animals during the whole experiment. (**g** to **h**): Locomotor and vertical activity. Diet and Photoperiod effects were analysed by 2-way ANOVA followed by LSD post hoc test ($p<0.05$). * indicates diet effect comparing STD and CAF-fed rats into each photoperiod conditions. Not significant photoperiod effects were found. Data are plotted as the mean ± SD (n=7-8). L6: 6h light/18h darkness; L12: 12h light/12h darkness; L18: 18h light/6h darkness; STD: standard diet; CAF: cafeteria diet.

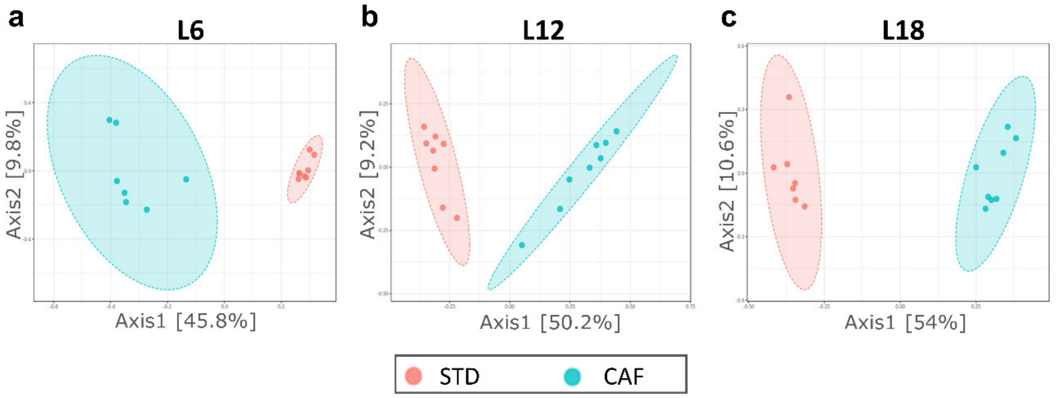

Figure S2. Effect of diet on the β-diversity in STD and CAF groups. β-diversity based on Bray-Curtis distances and visualized by a principle coordinates analysis (PCoA) 2D plot of Diet effect. (PERMANOVA test, p<0.001). (n=7-8). L6: 6h light/18h darkness; L12: 12h light/12h darkness; L18: 18h light/6h darkness; STD: standard diet; CAF: cafeteria diet.

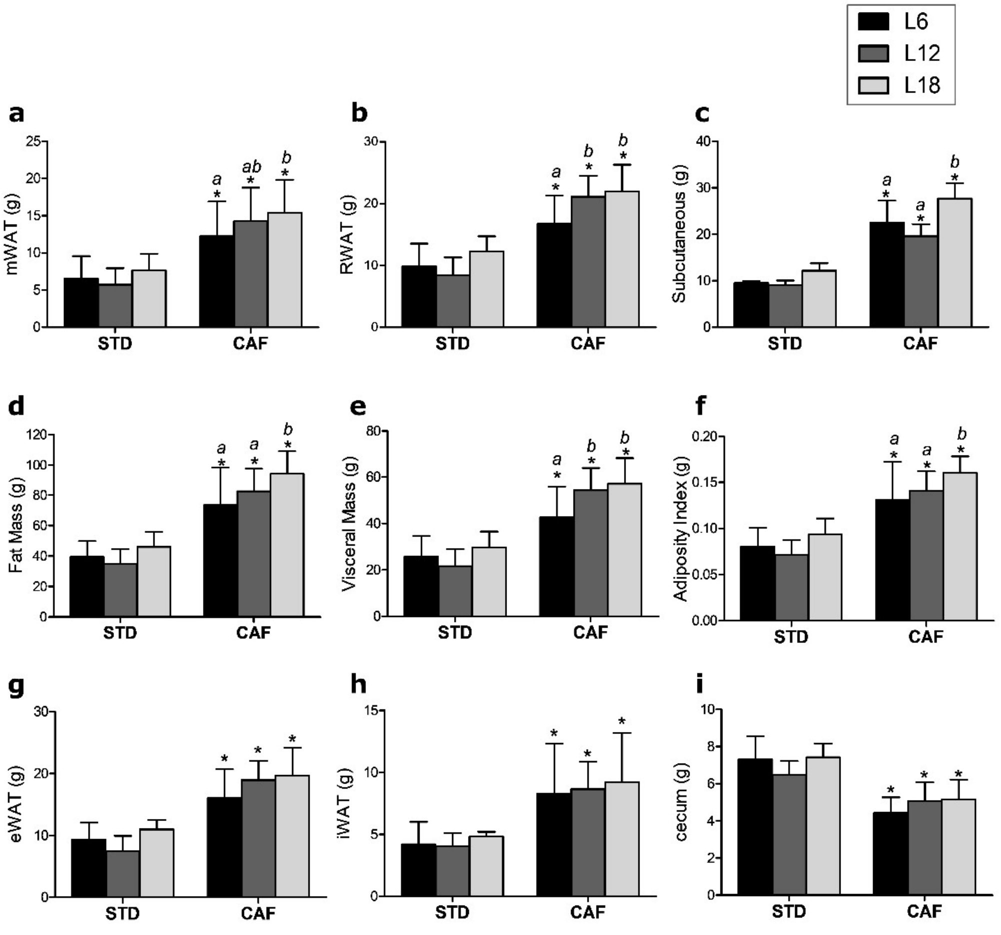

Figure S3. Effects of photoperiod on white adipose tissue depots and cecum weight. (**a** to **f**): White adipose tissue depots affected by photoperiods: mesenteric white adipose tissue (mWAT), retroperitoneal (RWAT), Subcutaneous, fat mass, visceral mass and adiposity index. (**g** to **i**): White adipose tissue depots and Cecum affected only by diet: epididymal white adipose tissue (eWAT), inguinal white adipose tissue (iWAT) and cecum; Diet and Photoperiod effects were analysed by 2-way ANOVA followed by LSD post hoc test (p<0.05). * and *ab* indicate diet and photoperiod effect respectively. Data are plotted as the mean±SD (n=7-8). L6: 6h light/18h darkness; L12: 12h light/12h darkness; L18: 18h light/6h darkness; STD: standard diet; CAF: cafeteria diet.

Table S1. Relative abundance at phylum level of STD- and CAF-fed rats under the three different photoperiods conditions (L6, L12 and L18)

Bacteria at Phylum level	Photoperiod	STD Median (Q1-Q3)[a]	P-value Ph STD[b]	CAF Median (Q1-Q3)[a]	P-value Ph CAF[b]	P-value DIET[c]
**Other**	L6	0.002(0.001-0.003)	0.109	0.02(0.0054-0.03)**	0.292	0.008
	L12	8.71e-04(4.83e-04-0.002)		0.01(0.004-0.01)**		0.001
	L18	0.002(0.002-0.006)		0.006(0.005-0.008)*		0.049
**Actinobacteria**	L6	0.07(0.05-0.13)	0.408	0.08(0.04-0.18)	0.321	1.000
	L12	0.08(0.04-0.36)		0.05(0.05-0.06)		0.294
	L18	0.10(0.07-0.49)		0.06(0.03-0.06)*		0.021
**Bacteroidetes**	L6	43.71(25.91-50.17)	0.383	52.24(44.61-70.78)*	0.084	0.064
	L12	34.34(26.74-45.67)		53.86(46.92-61.94)**		0.006
	L18	46.78(31.68-56.16)		69.27(54.76-76.93)**		0.005
**Cyanobacteria**	L6	0.19(0.11-0.23)	0.075	0.51(0.17-0.97)	0.538	0.105
	L12	0.10(0.07-0.17)		0.30(0.23-0.42)**		0.002
	L18	0.18(0.14-0.28)		0.41(0.30-0.57)**		0.004
**Firmicutes**	L6	53.26(46.50-71.98)	0.383	42.02(23.69-49.75)*	0.072	0.021
	L12	63.49(52.40-71.10)		40.02(32.87-48.83)**		0.003
	L18	50.65(39.90-61.40)		24.51(18.53-37.85)**		0.005
**Proteobacteria**	L6	1.10(0.69-1.33)	0.553	3.02(2.22-3.71)**	0.418	0.001
	L12	0.96(0.53-1.28)		3.26(1.89-3.98)**		0.002
	L18	1.07(0.86-1.21)		4.23(2.06-5.26)**		0.004
**Tenericutes**	L6	0.44(0.12-0.69)	0.092	7.66e-04(2.64e-04-0.009)**	0.846	0.001
	L12	0.53(0.30-0.60)		0.001(6.53e-05-0.003)**		0.001
	L18	1.21(0.35-1.66)		7.92e-04(3.27e-04-0.002)**		0.001
**Verrucomicrobia**	L6	0.54(0.39-0.83)	0.167	1.92(1.23-2.26)*	0.722	0.011
	L12	0.30(0.14-0.47)		1.63(0.71-3.76)**		0.002
	L18	0.54(0.36-0.65)		1.13(0.92-1.83)**		0.002
**Ratio Firmicutes to Bacteroidetes**	L6	1.22(0.93-2.98)	0.383	0.80(0.33-1.12)*	0.099	0.037
	L12	1.90(1.16-2.66)		0.75(0.52-1.04)**		0.006
	L18	1.08(0.71-1.94)		0.35(0.24-0.70)**		0.005

[a]Data shown as median (first and third quartile) in percentage of relative abundance (n=7-8).

[b]P-value by Kruskal-Wallis test comparing photoperiods in STD- or in CAF-fed rats. No photoperiod effect was found at phylum level (P>0.05).

[c]P-value by U-Mann Whitney to evaluate diet effect: * indicates diet effect when comparing STD and CAF-fed rats by U-Mann Whitney test into each photoperiod. *P<0.05; **P<0.01.

L6: 6h light/18h darkness; L12: 12h light/12h darkness; L18: 18h light/6h darkness; STD: standard diet; CAF: cafeteria diet.

Table S2. Significant Photoperiod and Diet effect at Genus level

Bacteria at Genus level	Photoperiod	STD	P-value Ph STD[b]	CAF	P-value Ph CAF[b]	P-value DIET[c]
		Median (Q1-Q3)[a]		Median (Q1-Q3)[a]		
Oscillospira	L6	9.94(7.81-12.9)	0.674	3.80(2.86-4.95)**a	0.015	0.001
	L12	10.7(7.89-11.6)		2.65(2.00-3.42)**ab		0.001
	L18	8.52(6.23-11.9)		2.03(1.77-2.63)**b		0.001
Bacteroides	L6	2.06(0.96-2.87)	0.856	5.77(3.97-12.4)***a*	0.004	0.001
	L12	1.45(0.87-2.60)		4.37(2.77-4.82)***a*		0.006
	L18	2.11(0.79-2.70)		10.1(8.22-15.9)***b*		0.001
Ruminococcus	L6	1.33(1.20-2.11)	0.820	0.47(0.17-0.98)**a*	0.014	0.015
	L12	1.60(1.33-1.97)		0.38(0.18-0.56)***a*		0.001
	L18	1.62(1.01-2.04)		0.14(0.10-0.17)***b*		0.001
Coprococcus	L6	0.82(0.46-1.04)	0.807	1.17(0.57-3.53)*a*	0.040	0.247
	L12	0.74(0.67-0.85)		3.06(1.91-4.57)***ab*		0.001
	L18	0.57(0.51-0.97)		4.12(2.23-5.57)***b*		0.001
Lactobacillus	L6	1.10(0.41-1.91)	0.642	0.69(0.53-1.53)*a*	0.025	0.908
	L12	1.39(0.59-2.72)		2.38(1.19-3.94)*b*		0.345
	L18	1.40(1.01-2.43)		0.64(0.17-2.16)*a*		0.298
Akkermansia	L6	0.54(0.39-0.86)	0.167	1.92(1.23-2.26)*	0.722	0.011
	L12	0.30(0.14-0.47)		1.63(0.71-3.76)**		0.002
	L18	0.54(0.36-0.65)		1.13(0.92-1.83)**		0.002
Parabacteroides	L6	0.63(0.26-0.79)	0.875	12.45(12.01-17.97)**	0.774	0.001
	L12	0.43(0.23-0.74)		16.87(5.55-19.77)**		0.002
	L18	0.47(0.32-0.87)		16.29(11.20-23.30)**		0.001
SMB53	L6	0.12(0.01-0.31)	0.940	7.99e-04(0-2.36e-03)**	0.625	0.004
	L12	0.09(0.06-0.17)		3.34e-04(5.31e-05-1.01e-03)**		0.001
	L18	0.07(0-0.42)		2.45e-04(5.91e-05-7.09e-04)**		0.001
Anaeroplasma	L6	0.09(0.03-0.29)	0.964	2.64e-04(0-5.11e-04)**	0.151	0.005
	L12	0.16(0.01-0.41)		1.21e-04(0-7.75e-04)**		0.003
	L18	0.04(0-1.13)		5.75e-04(3.17e-04-1.70e-03)*		0.024
Blautia	L6	0.08(0.07-0.13)	0.215	8.68(5.19-17.1)**	0.610	0.001
	L12	0.13(0.09-0.19)		8.00(6.86-10.2)**		0.001
	L18	0.19(0.09-0.20)		7.48(3.27-11.6)**		0.001
Lactococcus	L6	0.12(0.05-0.16)	0.431	4.64e-03(1.24e-03-7.91e-03)**	0.483	0.001
	L12	0.06(0.04-0.11)		6.39e-03(2.39e-03-1.78e-02)**		0.002
	L18	0.09(0.07-0.30)		1.27e-02(2.96e-03-2.74e-02)**		0.001
Bifidobacterium	L6	0.02(0-0.09)	0.543	4.42e-03(5.27e-04-5.25e-02)	0.528	0.728
	L12	0.04(0.01-0.30)		2.87e-03(1.18e-03-6.38e-03)*		0.021
	L18	0.06(0.01-0.43)		1.59e-03(5.97e-04-1.00e-02)**		0.005

Anaerostipes	L6	0.06(0.04-0.08)	0.343	5.90e-04(0-2.51e-02)**	0.589	0.005
	L12	0.09(0.05-0.12)		6.58e-04(5.68e-05-4.10e-02)*		0.021
	L18	0.11(0.06-0.16)		3.43e-04(0-7.47e-04)**		0.001
Dehalobacterium	L6	0.05(0.03-0.07)	0.774	0.27(5.96e-02-0.46)*	0.162	0.021
	L12	0.07(0.05-0.09)		0.13(1.48e-02-0.20)		0.401
	L18	0.06(0.03-0.08)		9.50e-02(1.58e-03-0.15)		0.643
Dorea	L6	0.06(0.05-0.07)	0.589	0.12(3.32e-02-0.17)	0.270	0.355
	L12	0.06(0.05-0.10)		0.19(0.11-0.39)*		0.027
	L18	0.07(0.06-0.08)		0.28(0.10-0.39)*		0.021
Sutterella	L6	0.06(0.03-0.08)	0.190	1.05(0.89-1.59)**	0.976	0.001
	L12	0.04(0.02-0.13)		1.10(0.74-2.39)**		0.001
	L18	0.09(0.05-0.15)		1.24(0.50-2.55)**		0.001
Roseburia	L6	0.04(0.01-0.06)	0.278	0.30(0.02-0.84)*	0.475	0.049
	L12	0.08(0.03-0.10)		0.12(0.06-0.24)		0.141
	L18	0.06(0.03-0.11)		0.21(0.07-0.27)		0.083
Clostridium	L6	0.01(0.01-0.03)	0.564	5.60e-03(1.53e-03-1.50e-02)	0.059	0.083
	L12	0.01(0.01-0.03)		2.14e-03(4.80e-04-8.64e-03)*		0.012
	L18	0.01(0.01-0.02)		8.93e-04(2.43e-04-1.53e-03)**		0.001
Lachnospira	L6	0.01(0-0.04)*a*	0.002	1.02e-03(2.64e-04-6.29e-03)*	0.344	0.015
	L12	0.01(0.01-0.02)*a*		4.43e-03(1.47e-03-8.48e-03)		0.115
	L18	1.10e-03(7.80e-04-2.03e-03)*b*		3.81e-03(1.36e-03-6.98e-03)*		0.037
Bilophila	L6	7.85e-03(4.31e-03-1.04e-02)	0.653	3.20e-03(7.66e-04-8.28e-03)	0.201	0.064
	L12	6.50e-03(2.53e-03-1.04e-02)		1.29e-03(2.21e-04-2.77e-03)*		0.015
	L18	4.62e-03(3.05e-03-9.62e-03)		9.70e-04(2.41e-04-2.04e-03)**		0.003
Streptococcus	L6	5.49e-03(3.43e-03-1.19e-02)*ab*	0.010	1.02e-02(5.80e-03-1.66e-02)	0.834	0.298
	L12	3.00e-03(1.36e-03-5.00e-03)*a*		1.00e-02(7.10e-03-1.29e-02)**		0.003
	L18	1.30e-02(7.51e-03-1.74e-02)*b*		1.03e-02(5.91e-03-1.46e-02)		0.298
Moryella	L6	3.59e-03(1.99e-03-6.70e-03)*a*	0.033	8.28e-04(6.00e-04-1.32e-03)**	0.092	0.008
	L12	9.51e-03(7.90e-03-1.27e-02)*ab*		1.01e-03(9.23e-04-1.11e-03)**		0.001
	L18	8.04e-03(4.19e-03-1.20e-02)*b*		1.89e-03(1.19e-03-2.08e-03)**		0.001
Aggregatibacter	L6	3.91e-03(8.89e-04-4.87e-03)*ab*	0.034	6.21e-03(3.40e-03-1.33e-02)	0.739	0.132
	L12	1.81e-03(5.51e-05-2.97e-03)*a*		5.00e-03(1.89e-03-1.09e-02)		0.074
	L18	7.39e-03(3.90e-03-2.16e-02)*b*		1.25e-02(2.96e-03-2.02e-02)		0.643
Anaerotruncus	L6	2.40e-03(1.40e-03-2.99e-03)*a*	0.006	1.28e-02(5.00e-03-8.49e-02)**	0.431	0.005
	L12	2.62e-03(1.69e-03-4.13e-03)*a*		3.68e-02(1.82e-02-6.35e-02)**		0.003
	L18	1.41e-02(1.12e-02-3.99e-02)*b*		1.98e-02(1.32e-02-4.27e-02)		0.643
Allobaculum	L6	8.24e-04(2.79e-04-4.74e-03)	0.492	8.86e-02(7.66e-04-0.18)*a*	0.044	0.064
	L12	6.53e-04(2.00e-04-3.45e-03)		3.16e-03(5.99e-04-6.75e-02)*ab*		0.093
	L18	5.78e-04(0-1.61e-03)		4.53e-04(2.39e-04-1.70e-03)*b*		0.816

	L6	1.89e-03(6.90e-04-3.64e-03)*ab*		6.63e-04(4.49e-04-8.28e-04)*a*		0.132
Epulopiscium	L12	2.29e-03(1.04e-03-4.44e-03)*a*	0.021	2.60e-03(1.22e-03-4.22e-03)*b*	0.027	0.916
	L18	2.20e-04(0-7.62e-04)*b*		7.85e-04(5.32e-05-1.66e-03)*ab*		0.346
	L6	1.18e-03(9.56e-04-2.46e-03)		1.24e-03(4.49e-04-1.47e-03)		0.954
Rothia	L12	8.92e-04(2.81e-04-2.18e-03)	0.156	8.67e-04(5.15e-04-1.24e-03)	0.050	0.916
	L18	2.87e-03(1.16e-03-4.26e-03)		3.72e-04(6.33e-05-6.66e-04)**		0.001
	L6	2.11e-04(4.48e-05-1.24e-03)*a*		9.99e-04(2.64e-04-1.53e-03)**a*		0.034
Enterobacter	L12	2.10e-04(0-4.30e-04)*a*	0.037	7.51e-04(2.47e-04-4.37e-03)**a*	0.009	0.035
	L18	3.21e-03(2.89e-04-7.06e-03)*b*		5.85e-03(3.36e-03-9.10e-03)*b*		0.105
	L6	5.37e-04(2.06e-04-9.26e-04)*a*		1.53e-03(4.49e-04-7.66e-03)		0.165
Shuttleworthia	L12	6.36e-04(2.26e-04-1.74e-03)*a*	0.008	1.22e-03(5.83e-04-2.21e-03)	0.875	0.270
	L18	0(0-0)*b*		1.30e-03(2.41e-04-5.48e-03)**		0.004
	L6	7.18e-04(4.17e-04-9.33e-04)*a*		1.39e-02(4.74e-03-2.04e-02)*		0.011
Xenorhabdus	L12	6.30e-04(4.47e-04-1.85e-03)*a*	0.018	1.32e-02(1.34e-03-0.15)*	0.884	0.012
	L18	1.75e-03(1.16e-03-5.15e-03)*b*		1.32e-02(3.54e-03-4.84e-02)		0.064
	L6	4.72e-04(2.73e-04-1.25e-03)*a*		4.00e-04(0-5.11e-04)		0.267
Comamonas	L12	0(0-0)*b*	0.009	0(0-0)	0.112	0.927
	L18	4.48e-04(2.54e-04-1.75e-03)*a*		2.16e-04(0-7.24e-04)		0.239
	L6	2.99e-04(2.05e-04-6.84e-04)		8.25e-03(0-1.38e-02)		0.245
Escherichia	L12	3.84e-04(5.09e-05-4.58e-04)	0.382	4.61e-03(5.74e-04-6.38e-02)*	0.328	0.012
	L18	8.75e-04(2.54e-04-2.69e-03)		1.24e-02(1.56e-03-3.15e-02)*		0.028
	L6	8.96e-05(0-8.23e-04)		1.02e-03(8.28e-04-1.66e-03)*		0.045
Anaerofustis	L12	3.01e-04(3.87e-05-4.32e-04)	0.955	1.66e-03(9.29e-04-2.66e-03)**	0.219	0.003
	L18	2.89e-04(0-7.62e-04)		8.84e-04(3.45e-04-1.47e-03)		0.063
	L6	8.96e-05(0-2.20e-04)		5.27e-04(3.32e-04-8.84e-04)**		0.007
Butyrivibrio	L12	2.04e-04(0-3.55e-04)	0.205	5.94e-04(2.95e-04-1.80e-03)*	0.761	0.030
	L18	0(0-0)		1.03e-03(3.20e-04-1.50e-03)**		0.005
	L6	0(0-1.89e-04)*a*		4.49e-04(2.64e-04-1.79e-03)**		0.005
Klebsiella	L12	1.77e-04(0-2.16e-04)*b*	0.013	1.26e-03(0-1.14e-02)	0.751	0.178
	L18	5.20e-04(2.20e-04-8.75e-04)*ab*		1.23e-03(4.87e-04-3.82e-03)		0.247
	L6	0(0-2.24e-05)a		2.64e-04(0-1.33e-03)*		0.046
Morganella	L12	9.94e-05(0-3.82e-04)b	0.024	0(0-6.63e-04)	0.340	0.469
	L18	2.60e-04(2.20e-04-4.48e-04)ab		2.50e-04(5.41e-05-1.19e-03)		1.000

[a]Data shown as median (first and third quartile) in percentage of relative abundance (n=7-8).

[b]P-value by Kruskal-Wallis test comparing photoperiods in STD- or in CAF-fed rats. *ab* letters indicate Photoperiod effect analyzed by Kruskal-Wallis test followed by Bonferroni *p*-values adjustment: p<0.016.

[c]P-value by U-Mann Whitney to evaluate diet effect. * indicates diet effect when comparing STD- and CAF-fed rats into each photoperiod by U-Mann Whitney test: *P<0.05. **P<0.01.

L6: 6h light/18h darkness; L12: 12h light/12h darkness; L18: 18h light/6h darkness; STD: standard diet; CAF: cafeteria diet.

Table S3. Significant Spearman's correlations between Body weight gain and fat parameters with the relative abundance bacteria at different taxonomic levels

Body weight gain and Fat parameters	Bacteria	rho[a]	P-value[b]	FDR[b]
	PHYLUM			
Fat mass	Proteobacteria	0.743	3.25E-09	0.001
RWAT	Proteobacteria	0.740	4.16E-09	0.002
Visceral fat	Proteobacteria	0.734	6.55E-09	0.002
Adiposity Index	Proteobacteria	0.731	8.14E-09	0.003
Gain weight (g)	Proteobacteria	0.723	1.38E-08	0.004
iWAT	Proteobacteria	0.719	1.76E-08	0.005
eWAT	Proteobacteria	0.700	6.06E-08	0.006
Fat mass	Tenericutes	-0.697	7.45E-08	0.006
mWAT	Proteobacteria	0.689	1.21E-07	0.007
iWAT	Tenericutes	-0.677	2.34E-07	0.008
Adiposity Index	Tenericutes	-0.676	2.60E-07	0.009
Visceral fat	Tenericutes	-0.672	3.16E-07	0.010
RWAT	Tenericutes	-0.663	5.13E-07	0.010
eWAT	Tenericutes	-0.663	5.19E-07	0.011
iWAT	Cyanobacteria	0.648	1.10E-06	0.012
Gain weight (g)	Tenericutes	-0.643	1.45E-06	0.013
eWAT	Verrucomicrobia	0.629	2.85E-06	0.013
Subcutaneous	Proteobacteria	0.621	4.11E-06	0.014
mWAT	Tenericutes	-0.609	7.21E-06	0.015
iWAT	Verrucomicrobia	0.607	7.60E-06	0.016
Fat mass	Cyanobacteria	0.591	1.52E-05	0.017
Visceral fat	Verrucomicrobia	0.586	1.90E-05	0.017
Fat mass	Verrucomicrobia	0.585	1.94E-05	0.018
eWAT	Cyanobacteria	0.579	2.46E-05	0.019
eWAT	Firmicutes	-0.572	3.22E-05	0.020
mWAT	Cyanobacteria	0.570	3.52E-05	0.021
Adiposity Index	Verrucomicrobia	0.567	4.03E-05	0.021
Adiposity Index	Cyanobacteria	0.564	4.45E-05	0.022
RWAT	Cyanobacteria	0.564	4.48E-05	0.023
RWAT	Verrucomicrobia	0.562	4.82E-05	0.024
mWAT	Verrucomicrobia	0.559	5.49E-05	0.025
Visceral fat	Cyanobacteria	0.558	5.51E-05	0.025
Gain weight (g)	Firmicutes	-0.550	7.53E-05	0.026
Gain weight (g)	Cyanobacteria	0.547	8.39E-05	0.027
Subcutaneous	Tenericutes	-0.542	9.92E-05	0.028
eWAT	Bacteroidetes	0.528	1.64E-04	0.029
Subcutaneous	Cyanobacteria	0.521	2.04E-04	0.029
Fat mass	Firmicutes	-0.519	2.18E-04	0.030
Visceral fat	Firmicutes	-0.514	2.55E-04	0.031
RWAT	Firmicutes	-0.509	3.05E-04	0.032
Gain weight (g)	Bacteroidetes	0.504	3.55E-04	0.033
Adiposity Index	Firmicutes	-0.500	4.00E-04	0.033
Gain weight (g)	Verrucomicrobia	0.477	8.11E-04	0.034
Visceral fat	Bacteroidetes	0.469	1.00E-03	0.035
Fat mass	Bacteroidetes	0.465	1.12E-03	0.036
Subcutaneous	Firmicutes	-0.457	1.42E-03	0.037
RWAT	Bacteroidetes	0.450	1.72E-03	0.037
Adiposity Index	Bacteroidetes	0.448	1.81E-03	0.038
Subcutaneous	Verrucomicrobia	0.445	1.95E-03	0.039
iWAT	Firmicutes	-0.443	2.06E-03	0.040
mWAT	Firmicutes	-0.438	2.31E-03	0.040
Subcutaneous	Bacteroidetes	0.406	5.15E-03	0.041
mWAT	Bacteroidetes	0.388	7.63E-03	0.042

iWAT	Bacteroidetes	0.364	1.28E-02	0.043
Visceral fat	Actinobacteria	-0.312	3.45E-02	0.044
eWAT	Actinobacteria	-0.311	3.57E-02	0.044
Fat mass	Actinobacteria	-0.302	4.11E-02	0.045
Adiposity Index	Actinobacteria	-0.284	5.57E-02	0.046
mWAT	Actinobacteria	-0.222	1.39E-01	0.047
RWAT	Actinobacteria	-0.221	1.40E-01	0.048
iWAT	Actinobacteria	-0.220	1.42E-01	0.048
Gain weight (g)	Actinobacteria	-0.210	1.61E-01	0.049
Subcutaneous	Actinobacteria	-0.167	2.67E-01	0.050
	CLASS			
Fat mass	Betaproteobacteria	0.774	2.93E-10	0.000
Adiposity Index	Betaproteobacteria	0.769	4.37E-10	0.001
Gain weight (g)	Betaproteobacteria	0.762	7.54E-10	0.001
RWAT	Betaproteobacteria	0.759	9.72E-10	0.002
Visceral fat	Betaproteobacteria	0.753	1.58E-09	0.002
eWAT	Betaproteobacteria	0.744	3.02E-09	0.003
iWAT	Betaproteobacteria	0.740	4.28E-09	0.003
eWAT	Erysipelotrichi	0.737	5.30E-09	0.003
Gain weight (g)	Erysipelotrichi	0.734	6.50E-09	0.004
mWAT	Betaproteobacteria	0.725	1.21E-08	0.004
Visceral fat	Erysipelotrichi	0.716	2.16E-08	0.005
Fat mass	Erysipelotrichi	0.712	2.82E-08	0.005
RWAT	Erysipelotrichi	0.704	4.67E-08	0.006
Subcutaneous	Betaproteobacteria	0.699	6.54E-08	0.006
Adiposity Index	Erysipelotrichi	0.697	7.32E-08	0.006
iWAT	Erysipelotrichi	0.692	9.89E-08	0.007
Fat mass	Mollicutes	-0.688	1.28E-07	0.007
Visceral fat	Mollicutes	-0.673	2.95E-07	0.008
eWAT	Mollicutes	-0.670	3.60E-07	0.008
mWAT	Erysipelotrichi	0.669	3.70E-07	0.009
Adiposity Index	Mollicutes	-0.669	3.81E-07	0.009
RWAT	Mollicutes	-0.666	4.45E-07	0.009
iWAT	Mollicutes	-0.655	7.86E-07	0.010
iWAT	4C0d_2	0.650	9.92E-07	0.010
Gain weight (g)	Mollicutes	-0.629	2.83E-06	0.011
eWAT	Verrucomicrobiae	0.629	2.85E-06	0.011
iWAT	Verrucomicrobiae	0.607	7.60E-06	0.012
eWAT	Clostridia	-0.604	8.88E-06	0.012
mWAT	Mollicutes	-0.604	8.89E-06	0.012
Gain weight (g)	Clostridia	-0.594	1.33E-05	0.013
Fat mass	4C0d_2	0.591	1.51E-05	0.013
Subcutaneous	Erysipelotrichi	0.590	1.60E-05	0.014
Visceral fat	Verrucomicrobiae	0.586	1.90E-05	0.014
Fat mass	Verrucomicrobiae	0.585	1.94E-05	0.015
eWAT	4C0d_2	0.581	2.35E-05	0.015
mWAT	4C0d_2	0.570	3.57E-05	0.015
Adiposity Index	Verrucomicrobiae	0.567	4.03E-05	0.016
RWAT	4C0d_2	0.565	4.36E-05	0.016
Adiposity Index	4C0d_2	0.564	4.51E-05	0.017
RWAT	Verrucomicrobiae	0.562	4.82E-05	0.017
Visceral fat	4C0d_2	0.559	5.41E-05	0.018
mWAT	Verrucomicrobiae	0.559	5.49E-05	0.018
Fat mass	Clostridia	-0.555	6.18E-05	0.018
Visceral fat	Clostridia	-0.553	6.61E-05	0.019

Gain weight (g)	4C0d_2	0.548	7.99E-05	0.019
Subcutaneous	Mollicutes	-0.542	9.88E-05	0.020
RWAT	Clostridia	-0.538	1.17E-04	0.020
Adiposity Index	Clostridia	-0.535	1.29E-04	0.021
eWAT	Bacteroidia	0.528	1.64E-04	0.021
Fat mass	Actinobacteria	-0.524	1.86E-04	0.021
Subcutaneous	4C0d_2	0.520	2.13E-04	0.022
eWAT	Actinobacteria	-0.518	2.27E-04	0.022
Visceral fat	Actinobacteria	-0.509	3.03E-04	0.023
Gain weight (g)	Bacteroidia	0.504	3.55E-04	0.023
Adiposity Index	Actinobacteria	-0.501	3.88E-04	0.024
Subcutaneous	Clostridia	-0.489	5.62E-04	0.024
mWAT	Clostridia	-0.482	6.90E-04	0.024
iWAT	Clostridia	-0.481	7.10E-04	0.025
Gain weight (g)	Verrucomicrobiae	0.477	8.11E-04	0.025
Visceral fat	Bacteroidia	0.469	1.00E-03	0.026
Fat mass	Bacteroidia	0.465	1.12E-03	0.026
RWAT	Bacteroidia	0.450	1.72E-03	0.026
Adiposity Index	Bacteroidia	0.448	1.81E-03	0.027
Subcutaneous	Verrucomicrobiae	0.445	1.95E-03	0.027
iWAT	Deltaproteobacteria	0.438	2.33E-03	0.028
RWAT	Actinobacteria	-0.428	2.98E-03	0.028
iWAT	Actinobacteria	-0.425	3.21E-03	0.029
Gain weight (g)	Actinobacteria	-0.419	3.79E-03	0.029
mWAT	Actinobacteria	-0.407	5.05E-03	0.029
Subcutaneous	Bacteroidia	0.406	5.15E-03	0.030
Subcutaneous	Actinobacteria	-0.405	5.22E-03	0.030
mWAT	Bacteroidia	0.388	7.63E-03	0.031
iWAT	Bacteroidia	0.364	1.28E-02	0.031
Visceral fat	Gammaproteobacteria	0.352	1.63E-02	0.032
eWAT	Alphaproteobacteria	0.350	1.70E-02	0.032
Subcutaneous	Alphaproteobacteria	0.341	2.02E-02	0.032
RWAT	Alphaproteobacteria	0.334	2.33E-02	0.033
RWAT	Deltaproteobacteria	0.321	2.94E-02	0.033
mWAT	Gammaproteobacteria	0.318	3.12E-02	0.034
	ORDER			
Fat mass	Burkholderiales	0.773	3.00E-10	3.70E-04
Adiposity Index	Burkholderiales	0.768	4.51E-10	0.001
Gain weight (g)	Burkholderiales	0.764	6.68E-10	0.001
RWAT	Burkholderiales	0.760	9.25E-10	0.001
Visceral fat	Burkholderiales	0.753	1.55E-09	0.002
eWAT	Burkholderiales	0.745	2.99E-09	0.002
iWAT	Burkholderiales	0.737	5.08E-09	0.003
eWAT	Erysipelotrichales	0.737	5.30E-09	0.003
Gain weight (g)	Erysipelotrichales	0.734	6.50E-09	0.003
mWAT	Burkholderiales	0.725	1.20E-08	0.004
Visceral fat	Erysipelotrichales	0.716	2.16E-08	0.004
Fat mass	Erysipelotrichales	0.712	2.82E-08	0.004
RWAT	Erysipelotrichales	0.704	4.67E-08	0.005

Subcutaneous	Burkholderiales	0.698	7.10E-08	0.005
Adiposity Index	Erysipelotrichales	0.697	7.32E-08	0.006
iWAT	Erysipelotrichales	0.692	9.89E-08	0.006
RWAT	Turicibacterales	-0.685	1.48E-07	0.006
eWAT	Pseudomonadales	0.679	2.19E-07	0.007
eWAT	Turicibacterales	-0.674	2.89E-07	0.007
mWAT	Erysipelotrichales	0.669	3.70E-07	0.007
Visceral fat	Turicibacterales	-0.668	3.90E-07	0.008
Fat mass	Turicibacterales	-0.668	3.96E-07	0.008
mWAT	Turicibacterales	-0.657	7.02E-07	0.009
Adiposity Index	Turicibacterales	-0.652	9.38E-07	0.009
iWAT	YS2	0.650	9.92E-07	0.009
Visceral fat	Pseudomonadales	0.644	1.39E-06	0.010
eWAT	Verrucomicrobiales	0.629	2.85E-06	0.010
Fat mass	Pseudomonadales	0.618	4.66E-06	0.010
iWAT	Turicibacterales	-0.615	5.51E-06	0.011
iWAT	Verrucomicrobiales	0.607	7.60E-06	0.011
eWAT	Clostridiales	-0.604	8.88E-06	0.011
Gain weight (g)	Turicibacterales	-0.597	1.21E-05	0.012
Adiposity Index	Pseudomonadales	0.596	1.25E-05	0.012
Gain weight (g)	Clostridiales	-0.594	1.33E-05	0.013
Fat mass	YS2	0.591	1.51E-05	0.013
Subcutaneous	Erysipelotrichales	0.590	1.60E-05	0.013
Visceral fat	Verrucomicrobiales	0.586	1.90E-05	0.014
Fat mass	Verrucomicrobiales	0.585	1.94E-05	0.014
eWAT	YS2	0.581	2.35E-05	0.014
RWAT	Anaeroplasmatales	-0.579	2.50E-05	0.015
RWAT	Pseudomonadales	0.575	2.96E-05	0.015
mWAT	YS2	0.570	3.57E-05	0.016
Adiposity Index	Verrucomicrobiales	0.567	4.03E-05	0.016
RWAT	YS2	0.565	4.36E-05	0.016
mWAT	Pseudomonadales	0.564	4.50E-05	0.017
Adiposity Index	YS2	0.564	4.51E-05	0.017
Gain weight (g)	Pseudomonadales	0.563	4.66E-05	0.017
RWAT	Verrucomicrobiales	0.562	4.82E-05	0.018
Visceral fat	YS2	0.559	5.41E-05	0.018
mWAT	Verrucomicrobiales	0.559	5.49E-05	0.019
Fat mass	Clostridiales	-0.555	6.18E-05	0.019
Visceral fat	Clostridiales	-0.553	6.61E-05	0.019
Gain weight (g)	YS2	0.548	7.99E-05	0.020
RWAT	Clostridiales	-0.538	1.17E-04	0.020
Adiposity Index	Clostridiales	-0.535	1.29E-04	0.020
mWAT	Anaeroplasmatales	-0.530	1.53E-04	0.021
iWAT	Anaeroplasmatales	-0.529	1.57E-04	0.021
eWAT	Bacteroidales	0.528	1.64E-04	0.021
iWAT	Pseudomonadales	0.526	1.76E-04	0.022
Subcutaneous	YS2	0.520	2.13E-04	0.022
Fat mass	Anaeroplasmatales	-0.519	2.22E-04	0.023
Visceral fat	Anaeroplasmatales	-0.513	2.64E-04	0.023
Adiposity Index	Anaeroplasmatales	-0.510	2.97E-04	0.023
Gain weight (g)	Bacteroidales	0.504	3.55E-04	0.024
Subcutaneous	Pseudomonadales	0.502	3.78E-04	0.024
eWAT	Anaeroplasmatales	-0.499	4.17E-04	0.024
Subcutaneous	Clostridiales	-0.489	5.62E-04	0.025
Subcutaneous	Turicibacterales	-0.486	6.12E-04	0.025

mWAT	Clostridiales	-0.482	6.90E-04	0.026
iWAT	Clostridiales	-0.481	7.10E-04	0.026
Gain weight (g)	Verrucomicrobiales	0.477	8.11E-04	0.026
eWAT	Bifidobacteriales	-0.475	8.44E-04	0.027
Fat mass	Bifidobacteriales	-0.471	9.55E-04	0.027
Visceral fat	Bacteroidales	0.469	1.00E-03	0.027
Gain weight (g)	Anaeroplasmatales	-0.466	1.10E-03	0.028
Fat mass	Bacteroidales	0.465	1.12E-03	0.028
Visceral fat	Bifidobacteriales	-0.455	1.51E-03	0.029
RWAT	Bacteroidales	0.450	1.72E-03	0.029
Adiposity Index	Bacteroidales	0.448	1.81E-03	0.029
Adiposity Index	Bifidobacteriales	-0.445	1.92E-03	0.030
Subcutaneous	Verrucomicrobiales	0.445	1.95E-03	0.030
Fat mass	Actinomycetales	-0.424	3.31E-03	0.030
Subcutaneous	Bacteroidales	0.406	5.15E-03	0.031
Visceral fat	Actinomycetales	-0.399	6.04E-03	0.031
RWAT	Actinomycetales	-0.397	6.37E-03	0.031
Adiposity Index	Actinomycetales	-0.393	6.84E-03	0.032
iWAT	Bifidobacteriales	-0.389	7.58E-03	0.032
mWAT	Bacteroidales	0.388	7.63E-03	0.033
Gain weight (g)	Bifidobacteriales	-0.388	7.63E-03	0.033
RWAT	Bifidobacteriales	-0.375	1.03E-02	0.033
iWAT	Bacteroidales	0.364	1.28E-02	0.034
mWAT	Bifidobacteriales	-0.363	1.33E-02	0.034
Subcutaneous	Actinomycetales	-0.356	1.51E-02	0.034
Subcutaneous	Anaeroplasmatales	-0.355	1.54E-02	0.035
Subcutaneous	Bifidobacteriales	-0.355	1.55E-02	0.035
eWAT	Actinomycetales	-0.347	1.81E-02	0.036
mWAT	Actinomycetales	-0.345	1.89E-02	0.036
iWAT	Actinomycetales	-0.335	2.28E-02	0.036
Subcutaneous	Pasteurellales	0.321	2.95E-02	0.037
Visceral fat	Rhizobiales	-0.315	3.30E-02	0.037
	FAMILY			
Fat mass	Clostridiaceae	-0.807	1.28E-11	3.27E-04
Adiposity Index	Clostridiaceae	-0.791	5.93E-11	0.001
Visceral fat	Clostridiaceae	-0.786	9.38E-11	0.001
eWAT	Clostridiaceae	-0.786	9.82E-11	0.001
RWAT	Clostridiaceae	-0.755	1.38E-09	0.002
eWAT	Erysipelotrichaceae	0.737	5.30E-09	0.002
Fat mass	Ruminococcaceae	-0.735	6.05E-09	0.002
Gain weight (g)	Erysipelotrichaceae	0.734	6.50E-09	0.003
Visceral fat	Ruminococcaceae	-0.726	1.16E-08	0.003
Adiposity Index	Ruminococcaceae	-0.725	1.18E-08	0.003
Gain weight (g)	Lachnospiraceae	0.723	1.41E-08	0.004
Gain weight (g)	Clostridiaceae	-0.722	1.45E-08	0.004
mWAT	Clostridiaceae	-0.721	1.62E-08	0.004
Visceral fat	Erysipelotrichaceae	0.716	2.16E-08	0.005
Fat mass	Erysipelotrichaceae	0.712	2.82E-08	0.005
Gain weight (g)	Ruminococcaceae	-0.711	3.01E-08	0.005
iWAT	Ruminococcaceae	-0.711	3.08E-08	0.006
iWAT	Lachnospiraceae	0.711	3.13E-08	0.006
Visceral fat	Lachnospiraceae	0.709	3.60E-08	0.006
mWAT	Lachnospiraceae	0.709	3.61E-08	0.007
Fat mass	Lachnospiraceae	0.707	3.93E-08	0.007

RWAT	Ruminococcaceae	-0.706	4.11E-08	0.007
RWAT	Erysipelotrichaceae	0.704	4.67E-08	0.008
Visceral fat	Porphyromonadaceae	0.703	5.24E-08	0.008
RWAT	Lachnospiraceae	0.702	5.33E-08	0.008
eWAT	Ruminococcaceae	-0.697	7.32E-08	0.008
Adiposity Index	Erysipelotrichaceae	0.697	7.32E-08	0.009
iWAT	Clostridiaceae	-0.696	8.03E-08	0.009
iWAT	Erysipelotrichaceae	0.692	9.89E-08	0.009
Adiposity Index	Porphyromonadaceae	0.692	1.02E-07	0.010
Adiposity Index	Lachnospiraceae	0.691	1.07E-07	0.010
Fat mass	Porphyromonadaceae	0.690	1.16E-07	0.010
eWAT	Porphyromonadaceae	0.681	1.89E-07	0.011
eWAT	Lachnospiraceae	0.679	2.10E-07	0.011
RWAT	Bacteroidaceae	0.678	2.25E-07	0.011
Fat mass	Mogibacteriaceae	-0.674	2.83E-07	0.012
Gain weight (g)	Porphyromonadaceae	0.670	3.46E-07	0.012
mWAT	Erysipelotrichaceae	0.669	3.70E-07	0.012
RWAT	Porphyromonadaceae	0.667	4.17E-07	0.013
iWAT	Streptococcaceae	-0.666	4.46E-07	0.013
mWAT	Ruminococcaceae	-0.660	6.02E-07	0.013
Gain weight (g)	Bacteroidaceae	0.660	6.15E-07	0.014
eWAT	Bacteroidaceae	0.658	6.64E-07	0.014
eWAT	Moraxellaceae	0.657	7.26E-07	0.014
Fat mass	Bacteroidaceae	0.656	7.46E-07	0.015
Subcutaneous	Clostridiaceae	-0.647	1.20E-06	0.015
Adiposity Index	Bacteroidaceae	0.647	1.21E-06	0.015
mWAT	Bacteroidaceae	0.646	1.22E-06	0.016
Adiposity Index	Mogibacteriaceae	-0.641	1.60E-06	0.016
Gain weight (g)	Mogibacteriaceae	-0.641	1.62E-06	0.016
Visceral fat	Mogibacteriaceae	-0.640	1.64E-06	0.017
Visceral fat	Bacteroidaceae	0.640	1.67E-06	0.017
iWAT	Porphyromonadaceae	0.635	2.09E-06	0.017
Subcutaneous	Ruminococcaceae	-0.634	2.24E-06	0.018
eWAT	Verrucomicrobiaceae	0.629	2.85E-06	0.018
mWAT	Porphyromonadaceae	0.629	2.91E-06	0.018
eWAT	Mogibacteriaceae	-0.628	3.00E-06	0.019
Visceral fat	Moraxellaceae	0.627	3.10E-06	0.019
RWAT	Mogibacteriaceae	-0.622	3.95E-06	0.019
iWAT	Mogibacteriaceae	-0.619	4.46E-06	0.020
Gain weight (g)	Streptococcaceae	-0.616	5.07E-06	0.020
RWAT	Streptococcaceae	-0.614	5.63E-06	0.020
iWAT	Verrucomicrobiaceae	0.607	7.60E-06	0.021
Fat mass	Moraxellaceae	0.605	8.30E-06	0.021
Fat mass	Streptococcaceae	-0.604	8.88E-06	0.021
Subcutaneous	Erysipelotrichaceae	0.590	1.60E-05	0.022

Visceral fat	Verrucomicrobiaceae	0.586	1.90E-05	0.022
Fat mass	Verrucomicrobiaceae	0.585	1.94E-05	0.022
Adiposity Index	Moraxellaceae	0.585	1.95E-05	0.023
RWAT	Anaeroplasmataceae	-0.579	2.50E-05	0.023
Subcutaneous	Mogibacteriaceae	-0.579	2.53E-05	0.023
Subcutaneous	Porphyromonadaceae	0.578	2.55E-05	0.024
mWAT	Mogibacteriaceae	-0.574	3.02E-05	0.024
Visceral fat	Streptococcaceae	-0.574	3.03E-05	0.024
Adiposity Index	Streptococcaceae	-0.570	3.58E-05	0.025
Visceral fat	Rikenellaceae	-0.569	3.70E-05	0.025
Adiposity Index	Verrucomicrobiaceae	0.567	4.03E-05	0.025
eWAT	Streptococcaceae	-0.564	4.51E-05	0.025
RWAT	Verrucomicrobiaceae	0.562	4.82E-05	0.026
mWAT	Verrucomicrobiaceae	0.559	5.49E-05	0.026
RWAT	Moraxellaceae	0.558	5.52E-05	0.026
eWAT	Rikenellaceae	-0.555	6.18E-05	0.027
mWAT	Moraxellaceae	0.552	7.10E-05	0.027
iWAT	Bacteroidaceae	0.549	7.91E-05	0.027
Subcutaneous	Lachnospiraceae	0.548	8.02E-05	0.028
Subcutaneous	Bacteroidaceae	0.546	8.68E-05	0.028
mWAT	Streptococcaceae	-0.546	8.81E-05	0.028
Gain weight (g)	Moraxellaceae	0.542	1.01E-04	0.029
mWAT	Anaeroplasmataceae	-0.530	1.53E-04	0.029
iWAT	Anaeroplasmataceae	-0.529	1.57E-04	0.029
Fat mass	Rikenellaceae	-0.527	1.66E-04	0.030
iWAT	Moraxellaceae	0.523	1.90E-04	0.030
Gain weight (g)	Rikenellaceae	-0.521	2.02E-04	0.030
Fat mass	Anaeroplasmataceae	-0.519	2.22E-04	0.031
RWAT	Rikenellaceae	-0.514	2.60E-04	0.031
Visceral fat	Anaeroplasmataceae	-0.513	2.64E-04	0.031
Adiposity Index	Anaeroplasmataceae	-0.510	2.97E-04	0.032
Adiposity Index	Rikenellaceae	-0.501	3.93E-04	0.032
eWAT	Anaeroplasmataceae	-0.499	4.17E-04	0.032
Subcutaneous	Moraxellaceae	0.493	5.07E-04	0.033
mWAT	Rikenellaceae	-0.491	5.34E-04	0.033
Gain weight (g)	Verrucomicrobiaceae	0.477	8.11E-04	0.033
eWAT	Bifidobacteriaceae	-0.475	8.44E-04	0.034
Subcutaneous	Streptococcaceae	-0.473	8.99E-04	0.034
Fat mass	Bifidobacteriaceae	-0.471	9.55E-04	0.034
Gain weight (g)	Anaeroplasmataceae	-0.466	1.10E-03	0.035

iWAT	Rikenellaceae	-0.459	1.35E-03	0.035
Visceral fat	Bifidobacteriaceae	-0.455	1.51E-03	0.035
Adiposity Index	Bifidobacteriaceae	-0.445	1.92E-03	0.036
Subcutaneous	Verrucomicrobiaceae	0.445	1.95E-03	0.036
Subcutaneous	Rikenellaceae	-0.420	3.65E-03	0.036
iWAT	Bifidobacteriaceae	-0.389	7.58E-03	0.037
Gain weight (g)	Bifidobacteriaceae	-0.388	7.63E-03	0.037
RWAT	Bifidobacteriaceae	-0.375	1.03E-02	0.037
iWAT	Christensenellaceae	-0.374	1.03E-02	0.038
mWAT	Bifidobacteriaceae	-0.363	1.33E-02	0.038
Subcutaneous	Anaeroplasmataceae	-0.355	1.54E-02	0.038
Subcutaneous	Bifidobacteriaceae	-0.355	1.55E-02	0.039
Adiposity Index	Christensenellaceae	-0.338	2.18E-02	0.039
Adiposity Index	Prevotellaceae	-0.336	2.25E-02	0.039
RWAT	Dehalobacteriaceae	0.335	2.29E-02	0.040
Subcutaneous	Pasteurellaceae	0.321	2.95E-02	0.040
Fat mass	Dehalobacteriaceae	0.319	3.07E-02	0.040
mWAT	Prevotellaceae	-0.317	3.21E-02	0.041
iWAT	Dehalobacteriaceae	0.316	3.24E-02	0.041
Subcutaneous	Prevotellaceae	-0.316	3.27E-02	0.041
Fat mass	Christensenellaceae	-0.313	3.39E-02	0.042
Fat mass	Prevotellaceae	-0.306	3.88E-02	0.042
Adiposity Index	Pasteurellaceae	0.302	4.10E-02	0.042
	GENUS			
eWAT	SMB53	-0.755	1.30E-09	1.54E-04
Gain weight (g)	Oscillospira	-0.732	7.57E-09	3.09E-04
Fat mass	SMB53	-0.729	9.16E-09	4.63E-04
Visceral fat	SMB53	-0.725	1.25E-08	0.001
eWAT	Oscillospira	-0.717	2.12E-08	0.001
Fat mass	Oscillospira	-0.717	2.14E-08	0.001
Visceral fat	Oscillospira	-0.714	2.46E-08	0.001
eWAT	Clostridium	-0.711	3.05E-08	0.001
Adiposity Index	SMB53	-0.707	3.96E-08	0.001
iWAT	Lactococcus	-0.706	4.11E-08	0.002
Adiposity Index	Clostridium	-0.705	4.56E-08	0.002
Adiposity Index	Oscillospira	-0.703	5.04E-08	0.002
Visceral fat	Clostridium	-0.701	5.66E-08	0.002
RWAT	SMB53	-0.701	5.80E-08	0.002
Fat mass	Clostridium	-0.701	5.83E-08	0.002
RWAT	Oscillospira	-0.698	6.85E-08	0.002
Gain weight (g)	Lactococcus	-0.688	1.24E-07	0.003
Fat mass	Anaerostipes	-0.683	1.72E-07	0.003
Fat mass	Lactococcus	-0.680	2.03E-07	0.003
Adiposity Index	Anaerostipes	-0.674	2.84E-07	0.003
Gain weight (g)	SMB53	-0.671	3.27E-07	0.003
RWAT	Lactococcus	-0.655	7.74E-07	0.003
Subcutaneous	Oscillospira	-0.654	8.26E-07	0.004
Visceral fat	Anaerostipes	-0.647	1.17E-06	0.004
eWAT	Anaerostipes	-0.644	1.35E-06	0.004
mWAT	SMB53	-0.642	1.53E-06	0.004
Adiposity Index	Lactococcus	-0.639	1.78E-06	0.004
eWAT	Ruminococcus	-0.638	1.88E-06	0.004

Adiposity Index	Ruminococcus	-0.637	1.89E-06	0.004
eWAT	Moryella	-0.634	2.25E-06	0.005
Fat mass	Ruminococcus	-0.629	2.88E-06	0.005
eWAT	Lactococcus	-0.628	2.97E-06	0.005
RWAT	Moryella	-0.627	3.11E-06	0.005
Visceral fat	Lactococcus	-0.626	3.27E-06	0.005
mWAT	Oscillospira	-0.625	3.38E-06	0.005
Gain weight (g)	Bilophila	-0.623	3.70E-06	0.006
Visceral fat	Ruminococcus	-0.620	4.28E-06	0.006
Visceral fat	Bilophila	-0.620	4.33E-06	0.006
RWAT	Anaerostipes	-0.618	4.76E-06	0.006
RWAT	Clostridium	-0.615	5.37E-06	0.006
Fat mass	Moryella	-0.614	5.72E-06	0.006
iWAT	Anaerostipes	-0.613	5.81E-06	0.006
Fat mass	Bilophila	-0.613	5.85E-06	0.007
Subcutaneous	SMB53	-0.612	6.15E-06	0.007
mWAT	Lactococcus	-0.610	6.89E-06	0.007
iWAT	SMB53	-0.605	8.47E-06	0.007
iWAT	Ruminococcus	-0.603	9.17E-06	0.007
eWAT	Bilophila	-0.602	9.57E-06	0.007
iWAT	Oscillospira	-0.601	1.02E-05	0.008
Gain weight (g)	Clostridium	-0.598	1.16E-05	0.008
Adiposity Index	Bilophila	-0.596	1.25E-05	0.008
Adiposity Index	Moryella	-0.593	1.39E-05	0.008
Gain weight (g)	Ruminococcus	-0.592	1.49E-05	0.008
mWAT	Clostridium	-0.591	1.55E-05	0.008
Visceral fat	Moryella	-0.591	1.56E-05	0.008
Subcutaneous	Anaerostipes	-0.588	1.72E-05	0.009
RWAT	Anaeroplasma	-0.579	2.50E-05	0.009
Gain weight (g)	Moryella	-0.576	2.83E-05	0.009
iWAT	Moryella	-0.576	2.84E-05	0.009
mWAT	Bilophila	-0.562	4.91E-05	0.009
RWAT	Bilophila	-0.560	5.27E-05	0.009
Subcutaneous	Lactococcus	-0.551	7.23E-05	0.010
mWAT	Anaerostipes	-0.548	8.10E-05	0.010
mWAT	Moryella	-0.548	8.16E-05	0.010
Subcutaneous	Clostridium	-0.538	1.15E-04	0.010
Gain weight (g)	Anaerostipes	-0.537	1.19E-04	0.010
RWAT	Ruminococcus	-0.535	1.26E-04	0.010
Subcutaneous	Bilophila	-0.534	1.30E-04	0.010
mWAT	Anaeroplasma	-0.530	1.53E-04	0.011
iWAT	Anaeroplasma	-0.529	1.57E-04	0.011
Subcutaneous	Moryella	-0.527	1.65E-04	0.011
Subcutaneous	Ruminococcus	-0.522	1.96E-04	0.011
iWAT	Bilophila	-0.519	2.19E-04	0.011
Fat mass	Anaeroplasma	-0.519	2.22E-04	0.011
Visceral fat	Anaeroplasma	-0.513	2.64E-04	0.012
Adiposity Index	Anaeroplasma	-0.510	2.97E-04	0.012
Fat mass	Rothia	-0.507	3.27E-04	0.012
iWAT	Clostridium	-0.505	3.45E-04	0.012
Visceral fat	Rothia	-0.504	3.60E-04	0.012
mWAT	Ruminococcus	-0.500	3.99E-04	0.012
mWAT	Roseburia	0.502	3.79E-04	0.013
iWAT	Butyrivibrio	0.502	3.73E-04	0.013
RWAT	Xenorhabdus	0.504	3.60E-04	0.013

mWAT	Escherichia	0.510	2.99E-04	0.013
Gain weight (g)	Escherichia	0.510	2.95E-04	0.013
eWAT	Klebsiella	0.511	2.87E-04	0.013
mWAT	Xenorhabdus	0.517	2.31E-04	0.013
Visceral fat	Anaerofustis	0.518	2.30E-04	0.014
Fat mass	Anaerofustis	0.519	2.16E-04	0.014
Visceral fat	Klebsiella	0.521	2.07E-04	0.014
Adiposity Index	Enterobacter	0.521	2.06E-04	0.014
Fat mass	Enterobacter	0.522	2.00E-04	0.014
eWAT	Enterobacter	0.531	1.48E-04	0.014
mWAT	Butyrivibrio	0.534	1.30E-04	0.015
mWAT	Coprococcus	0.536	1.22E-04	0.015
RWAT	Anaerotruncus	0.541	1.05E-04	0.015
eWAT	Anaerotruncus	0.543	9.65E-05	0.015
Visceral fat	Anaerotruncus	0.544	9.24E-05	0.015
iWAT	Coprococcus	0.545	9.09E-05	0.015
Subcutaneous	Bacteroides	0.546	8.68E-05	0.015
Adiposity Index	Anaerofustis	0.548	8.12E-05	0.016
iWAT	Bacteroides	0.549	7.91E-05	0.016
iWAT	Escherichia	0.554	6.59E-05	0.016
Visceral fat	Enterobacter	0.554	6.53E-05	0.016
Subcutaneous	Butyrivibrio	0.558	5.66E-05	0.016
Fat mass	Anaerotruncus	0.558	5.61E-05	0.016
Adiposity Index	Anaerotruncus	0.558	5.53E-05	0.017
mWAT	Akkermansia	0.559	5.49E-05	0.017
RWAT	Anaerofustis	0.560	5.16E-05	0.017
RWAT	Akkermansia	0.562	4.82E-05	0.017
Gain weight (g)	Dorea	0.563	4.71E-05	0.017
Adiposity Index	Butyrivibrio	0.566	4.14E-05	0.017
Adiposity Index	Akkermansia	0.567	4.03E-05	0.017
Visceral fat	Coprococcus	0.568	3.83E-05	0.018
RWAT	Butyrivibrio	0.568	3.81E-05	0.018
Visceral fat	Butyrivibrio	0.573	3.14E-05	0.018
Adiposity Index	Escherichia	0.577	2.68E-05	0.018
Fat mass	Escherichia	0.578	2.61E-05	0.018
Subcutaneous	Parabacteroides	0.578	2.55E-05	0.018
eWAT	Butyrivibrio	0.580	2.36E-05	0.019
Fat mass	Butyrivibrio	0.582	2.25E-05	0.019
eWAT	Coprococcus	0.585	1.97E-05	0.019
Fat mass	Akkermansia	0.585	1.94E-05	0.019
Visceral fat	Akkermansia	0.586	1.90E-05	0.019
RWAT	Coprococcus	0.586	1.85E-05	0.019
Subcutaneous	Coprococcus	0.590	1.62E-05	0.019
Fat mass	Xenorhabdus	0.590	1.62E-05	0.020
Gain weight (g)	Coprococcus	0.595	1.28E-05	0.020
Gain weight (g)	Butyrivibrio	0.602	9.79E-06	0.020
Adiposity Index	Xenorhabdus	0.604	8.74E-06	0.020
eWAT	Escherichia	0.605	8.27E-06	0.020
iWAT	Akkermansia	0.607	7.60E-06	0.020
Visceral fat	Escherichia	0.611	6.39E-06	0.021
Adiposity Index	Coprococcus	0.615	5.44E-06	0.021
Fat mass	Coprococcus	0.619	4.50E-06	0.021
mWAT	Parabacteroides	0.629	2.91E-06	0.021
eWAT	Akkermansia	0.629	2.85E-06	0.021
iWAT	Parabacteroides	0.635	2.09E-06	0.021

Visceral fat	Bacteroides	0.640	1.67E-06	0.021
Visceral fat	Xenorhabdus	0.646	1.27E-06	0.022
mWAT	Bacteroides	0.646	1.22E-06	0.022
Adiposity Index	Bacteroides	0.647	1.21E-06	0.022
Fat mass	Bacteroides	0.656	7.46E-07	0.022
eWAT	Bacteroides	0.658	6.64E-07	0.022
eWAT	Xenorhabdus	0.659	6.40E-07	0.022
Gain weight (g)	Bacteroides	0.660	6.15E-07	0.023
iWAT	Blautia	0.661	5.71E-07	0.023
RWAT	Parabacteroides	0.667	4.17E-07	0.023
Gain weight (g)	Parabacteroides	0.670	3.46E-07	0.023
RWAT	Bacteroides	0.678	2.25E-07	0.023
Subcutaneous	Blautia	0.678	2.20E-07	0.023
eWAT	Parabacteroides	0.681	1.89E-07	0.023
Fat mass	Parabacteroides	0.690	1.16E-07	0.024
Adiposity Index	Parabacteroides	0.692	1.02E-07	0.024
Subcutaneous	Sutterella	0.695	8.13E-08	0.024
Visceral fat	Parabacteroides	0.703	5.24E-08	0.024
mWAT	Blautia	0.710	3.22E-08	0.024
mWAT	Sutterella	0.724	1.26E-08	0.024
Gain weight (g)	Blautia	0.735	6.13E-09	0.025
eWAT	Blautia	0.736	5.44E-09	0.025
Adiposity Index	Blautia	0.740	4.35E-09	0.025
eWAT	Sutterella	0.741	3.94E-09	0.025
Visceral fat	Blautia	0.742	3.73E-09	0.025
iWAT	Sutterella	0.742	3.49E-09	0.025
Visceral fat	Sutterella	0.752	1.74E-09	0.025
Fat mass	Blautia	0.754	1.42E-09	0.026
RWAT	Sutterella	0.760	9.30E-10	0.026
Gain weight (g)	Sutterella	0.760	9.20E-10	0.026
RWAT	Blautia	0.767	5.13E-10	0.026
Adiposity Index	Sutterella	0.768	4.55E-10	0.026
Fat mass	Sutterella	0.773	3.19E-10	0.026
eWAT	Anaeroplasma	-0.499	4.17E-04	0.027
Adiposity Index	Rothia	-0.496	4.59E-04	0.027
eWAT	Bifidobacterium	-0.475	8.44E-04	0.027
Fat mass	Bifidobacterium	-0.471	9.55E-04	0.027
Gain weight (g)	Anaeroplasma	-0.466	1.10E-03	0.027
Visceral fat	Bifidobacterium	-0.455	1.51E-03	0.027
Subcutaneous	Rothia	-0.452	1.61E-03	0.027
Adiposity Index	Bifidobacterium	-0.445	1.92E-03	0.028
eWAT	Rothia	-0.433	2.62E-03	0.028
RWAT	Rothia	-0.419	3.75E-03	0.028
mWAT	Rothia	-0.414	4.27E-03	0.028
Gain weight (g)	Rothia	-0.397	6.35E-03	0.028
iWAT	Bifidobacterium	-0.389	7.58E-03	0.028
Gain weight (g)	Bifidobacterium	-0.388	7.63E-03	0.029
RWAT	Bifidobacterium	-0.375	1.03E-02	0.029
mWAT	Bifidobacterium	-0.363	1.33E-02	0.029
Subcutaneous	Anaeroplasma	-0.355	1.54E-02	0.029
Subcutaneous	Bifidobacterium	-0.355	1.55E-02	0.029
iWAT	Rothia	-0.335	2.27E-02	0.029
Visceral fat	Dehalobacterium	0.297	4.50E-02	0.029
mWAT	Dehalobacterium	0.302	4.15E-02	0.030
Subcutaneous	Streptococcus	0.308	3.72E-02	0.030

Subcutaneous	Aggregatibacter	0.311	3.55E-02	0.030
eWAT	Allobaculum	0.313	3.44E-02	0.030
eWAT	Streptococcus	0.314	3.37E-02	0.030
eWAT	Dehalobacterium	0.315	3.30E-02	0.030
Subcutaneous	Dehalobacterium	0.316	3.22E-02	0.031
Adiposity Index	Dehalobacterium	0.322	2.89E-02	0.031
iWAT	Roseburia	0.332	2.41E-02	0.031
Visceral fat	Shuttleworthia	0.335	2.28E-02	0.031
Subcutaneous	Shuttleworthia	0.337	2.19E-02	0.031
mWAT	Allobaculum	0.341	2.05E-02	0.031
iWAT	Allobaculum	0.343	1.97E-02	0.031
eWAT	Roseburia	0.351	1.69E-02	0.032
Subcutaneous	Klebsiella	0.355	1.55E-02	0.032
Fat mass	Shuttleworthia	0.355	1.53E-02	0.032
iWAT	Dehalobacterium	0.357	1.49E-02	0.032
Fat mass	Dehalobacterium	0.359	1.43E-02	0.032
Gain weight (g)	Allobaculum	0.365	1.26E-02	0.032
iWAT	Klebsiella	0.367	1.22E-02	0.033
RWAT	Dehalobacterium	0.370	1.13E-02	0.033
Adiposity Index	Shuttleworthia	0.372	1.08E-02	0.033
Gain weight (g)	Anaerofustis	0.378	9.70E-03	0.033
Gain weight (g)	Klebsiella	0.382	8.76E-03	0.033
Subcutaneous	Escherichia	0.392	7.11E-03	0.033
Subcutaneous	Roseburia	0.392	6.99E-03	0.033
Visceral fat	Roseburia	0.397	6.23E-03	0.034
iWAT	Enterobacter	0.399	6.04E-03	0.034
Adiposity Index	Roseburia	0.405	5.30E-03	0.034
eWAT	Dorea	0.411	4.58E-03	0.034
Subcutaneous	Enterobacter	0.414	4.25E-03	0.034
RWAT	Klebsiella	0.416	4.05E-03	0.034
Fat mass	Roseburia	0.417	3.94E-03	0.035
iWAT	Xenorhabdus	0.421	3.56E-03	0.035
mWAT	Klebsiella	0.435	2.51E-03	0.035
mWAT	Anaerofustis	0.435	2.49E-03	0.035
Gain weight (g)	Anaerotruncus	0.444	1.97E-03	0.035
Subcutaneous	Dorea	0.445	1.96E-03	0.035
Subcutaneous	Akkermansia	0.445	1.95E-03	0.035
RWAT	Enterobacter	0.451	1.64E-03	0.036
RWAT	Roseburia	0.456	1.44E-03	0.036
RWAT	Escherichia	0.456	1.44E-03	0.036
Gain weight (g)	Roseburia	0.459	1.34E-03	0.036
Adiposity Index	Dorea	0.461	1.27E-03	0.036
iWAT	Dorea	0.461	1.27E-03	0.036
Subcutaneous	Xenorhabdus	0.462	1.23E-03	0.037
Subcutaneous	Anaerofustis	0.462	1.23E-03	0.037
Gain weight (g)	Enterobacter	0.464	1.17E-03	0.037
Visceral fat	Dorea	0.464	1.17E-03	0.037
mWAT	Enterobacter	0.470	9.69E-04	0.037
mWAT	Anaerotruncus	0.472	9.21E-04	0.037
Fat mass	Dorea	0.474	8.71E-04	0.038
Gain weight (g)	Akkermansia	0.477	8.11E-04	0.038
iWAT	Anaerofustis	0.477	7.97E-04	0.038
Fat mass	Klebsiella	0.479	7.62E-04	0.038
Gain weight (g)	Xenorhabdus	0.485	6.42E-04	0.038
mWAT	Dorea	0.486	6.11E-04	0.038

iWAT	Anaerotruncus	0.486	6.09E-04	0.038
Adiposity Index	Klebsiella	0.493	4.98E-04	0.039
RWAT	Dorea	0.494	4.86E-04	0.039
eWAT	Anaerofustis	0.498	4.34E-04	0.039
Subcutaneous	Anaerotruncus	0.499	4.13E-04	0.039

[a]Spearman's rank-order correlation coefficient (rho).

[b]The correlations were significant when the P-value and FDR (False Disvory Ratio) was < 0.05. (n=7-8)
